# Longitudinal study on hippocampal subfields and glucose metabolism in early psychosis

**DOI:** 10.1038/s41537-024-00475-z

**Published:** 2024-07-31

**Authors:** Reetta-Liina Armio, Heikki Laurikainen, Tuula Ilonen, Maija Walta, Elina Sormunen, Arvi Tolvanen, Raimo K. R. Salokangas, Nikolaos Koutsouleris, Lauri Tuominen, Jarmo Hietala

**Affiliations:** 1 PET Centre, Turku University Hospital, 20520 Turku, Finland; 2 Department of Psychiatry, University of Turku, 20700 Turku, Finland; 3 Department of Psychiatry, Turku University Hospital, 20520 Turku, Finland; 4 Department of Psychiatry and Psychotherapy, Ludwig-Maximilian University, D-80336 Munich, Germany; 5 The Royal’s Institute of Mental Health Research, University of Ottawa, Ottawa, ON Canada; 6 Department of Psychiatry, Faculty of Medicine, University of Ottawa, Ottawa, ON Canada

**Keywords:** Psychosis, Neuroscience, Psychosis

## Abstract

Altered hippocampal morphology and metabolic pathology, but also hippocampal circuit dysfunction, are established phenomena seen in psychotic disorders. Thus, we tested whether hippocampal subfield volume deficits link with deviations in glucose metabolism commonly seen in early psychosis, and whether the glucose parameters or subfield volumes change during follow-up period using one-year longitudinal study design of 78 first-episode psychosis patients (FEP), 48 clinical high-risk patients (CHR) and 83 controls (CTR). We also tested whether hippocampal morphology and glucose metabolism relate to clinical outcome. Hippocampus subfields were segmented with Freesurfer from 3T MRI images and parameters of glucose metabolism were determined in fasting plasma samples. Hippocampal subfield volumes were consistently lower in FEPs, and findings were more robust in non-affective psychoses, with strongest decreases in CA1, molecular layer and hippocampal tail, and in hippocampal tail of CHRs, compared to CTRs. These morphometric differences remained stable at one-year follow-up. Both non-diabetic CHRs and FEPs had worse glucose parameters compared to CTRs at baseline. We found that, insulin levels and insulin resistance increased during the follow-up period only in CHR, effect being largest in the CHRs converting to psychosis, independent of exposure to antipsychotics. The worsening of insulin resistance was associated with deterioration of function and symptoms in CHR. The smaller volume of hippocampal tail was associated with higher plasma insulin and insulin resistance in FEPs, at the one-year follow-up. Our longitudinal study supports the view that temporospatial hippocampal subfield volume deficits are stable near the onset of first psychosis, being more robust in non-affective psychoses, but less prominent in the CHR group. Specific subfield defects were related to worsening glucose metabolism during the progression of psychosis, suggesting that hippocampus is part of the circuits regulating aberrant glucose metabolism in early psychosis. Worsening of glucose metabolism in CHR group was associated with worse clinical outcome measures indicating a need for heightened clinical attention to metabolic problems already in CHR.

## Introduction

Psychotic disorders have been consistently associated with altered brain morphology^1,2^ including the hippocampus^3–10^, as well as with glucose metabolism already in first episode of psychosis^11,12^. Also, hippocampal circuit dysfunction in psychotic disorders, such as schizophrenia, is supported by several studies on brain structure and function^13–15^. Meta-analyses^16,17^ have demonstrated that hippocampal volume reductions are present in first-episode psychosis, but it is less clear whether the same is true for the individuals with clinical high-risk for psychosis^18–21^. Based on recent longitudinal study, the subfield volumes remain stable during the early years after the onset of psychosis^22^. The data is inconsistent whether hippocampal morphology predicts the transition to psychosis in high-risk individuals^18,20,23^. Furthermore, it is not known whether these morphological changes are associated with glucose metabolism disturbances in FEP or CHR, or in relation to clinical outcomes.

Hippocampal circuits are traditionally linked to regulation of learning, memory, spatial processing, emotions and stress responses^24^. These behavioral domains are all afflicted to a varying degree in psychotic disorders^14^. In addition, several lines of research suggest that hippocampus regulates endocrine functions and glucose metabolism^25,26^. Also, diabetes associates with brain morphology^4^ including the volume of the hippocampus^27–31^. Further, an inverse association between insulin resistance and total hippocampal volume has been reported in healthy individuals^32^, but also between insulin resistance and specific subfield volumes in type 2 diabetes^33,34^. There is also abundant evidence of altered glucose homeostasis in psychoses^35–38^ which cannot be attributed solely to the effects of antipsychotic medications^11,39–41^. In fact, psychotic disorders show peripheral changes of both glucose and lipid metabolism^12,39,42^, and the regulation between hippocampal circuitries and peripheral metabolism seems to be bidirectional^25^.

Associations between fasting glucose or glycated hemoglobin, and clinical outcome have been studied in FEPs, but not insulin or insulin resistance^43^. Also, recent study^44^ showed that worsening of fasting glucose values was related to progression of psychotic illness in recent-onset psychosis. Only some longitudinal studies have been published on subfields and clinical outcome in CHR^10,23^ or first-episode schizophrenia^45^. However, these studies do not combine glucose metabolism parameters and subfield volumetry to investigate the clinical outcome trajectories, in contrast to our study. Given this evidence base it is important to study whether peripheral metabolic factors relate to alterations in brain morphology as well as to illness progression and clinical outcome, even as early as during the first episode of psychosis.

In this longitudinal study, we first tested whether patients with first-episode psychosis, clinical high-risk individuals or population controls have associations between altered hippocampal subfield or total hippocampal volumes and glucose parameters at the two time points during 1-year follow-up. Secondly, we tested whether subfield volumes or the measurements of glucose homeostasis change during the one-year follow-up in these groups. Thirdly, we tested whether the cross-sectional associations or the longitudinal changes in patient groups are related to clinical outcome trajectories, such as remission, functioning, and transition to psychosis. We hypothesized that the hippocampal volume reductions are associated with progressive glucose metabolism disturbances, and that the effect is most pronounced in patients with first-episode psychosis and in CHR patients who developed psychosis during follow-up.

## Methods

### Participants

#### Participants at baseline

The intent-to-study groups consisted of age- and sex-matched 88 first-episode psychosis patients (FEP), 56 clinical high-risk for psychosis patients (CHR) and 96 randomly selected general population controls (CTR) between 18 and 50 years of age. Patients were recruited from psychiatric services of the Hospital District of Southwest Finland. The general population control group was recruited from the same geographic area using a random sample of the national population register. The study protocol was approved by the Ethics Committee of the Hospital District of Southwest Finland and the study was conducted in accordance with the Declaration of Helsinki. Written informed assent and consent were given by from all the participants.

The clinical high-risk status was defined by the ultra-high-risk criteria: Attenuated Psychotic Symptoms (APS), Brief Limited Psychotic Symptoms, and Genetic risk and reduction of function assessed by the 3.0/5.0 version of the Structured Interview for Prodromal Syndromes (SIPS/SOPS). Psychotic and non-psychotic diagnoses of all participants were evaluated using the Structured Clinical Interview for DSM-IV disorders (SCID-I/NP) Eight population control participants who had a non-psychotic DSM-IV diagnosis, were included in the study. Diagnoses are listed in Supplementary table 1.

Participants with an IQ under 70, a significant somatic or neurological illness that might affect brain structure or function, earlier head injury with loss of consciousness for over five minutes, or alcohol dependence during the preceding 6 months were excluded.

At baseline, one FEP, two CHRs and five CTRs with neurological findings in MRI (Supplementary table 2), and two FEPs and one CHR scanned with incompatible MRI scan parameters (non-iso T1 sequence) were excluded. One control participant who was later found to be a 1st degree relative of a psychosis spectrum disorder patient was excluded. We also excluded two FEPs, two CHRs and one CTR due to excessive motion in their T1 weighted images. The hippocampal segmentation failed in 12 subjects (five FEPs, two CHRs, five CTRs), who were also excluded. Further, one control participant was excluded due to a CA3 volume being a statistical outlier (standard residual: (SD) = 3.5 ≥ ± 3.5 SD; statistical influence: Cook’s mean x^3^ = 0.005 x^3^ = 0.015; Cook’s distance = 0.033 > 0.015) and one CHR participant was excluded due to the whole hippocampus volume being a statistical outlier (standard residual: (SD) = −3.5 ≥ ± 3.5 SD; statistical influence: Cook’s mean x^3^ = 0.005 x^3^ = 0.015; Cook’s distance = 0.051 > 0.015).

Six FEPs, ten CHRs and eight CTRs did not participate in blood sampling at one of the two time points. From analyses related to glucose metabolism variables, two FEPs, one CHR and two CTRs with diabetes mellitus diagnosis (type 1), and three CHRs and one CTR whose insulin sample was affected by hemolysis were excluded. Further, one FEP with an outlier value of insulin at follow-up (standard residual: (SD) = 3.5 ≥ ± 4.6 SD; statistical influence: Cook’s mean x^3^ = 0.019 x^3^ = 0.057; Cook’s distance = 0.823 > 0.057) was excluded from analyses of the glucose parameters.

The final baseline sample consisted of 78 first-episode psychosis patients, 48 clinical high-risk for patients for psychosis patients and 83 randomly selected general population controls of similar age and sex. The baseline demographical comparisons are presented in Table 1. At the baseline, FEP group consisted of 56 non-affective psychoses (NAP) and 22 affective psychoses (AP).

**Table 1:** Demographic characteristics of the participants at baseline.

	Mean ± SD (n)			*p*-value * (t-value, df)		
	FEPs	CHRs	CTRs	FEP vs CTR	CHR vs CTR	FEP vs CHR
Age	26.9 ± 6.0 (78)	25.9 ± 6.1 (48)	27.7 ± 5.5 (83)	0.336 (−1.0, 159)	0.080 (−1.8, 129)	0.391 (0.9,124)
Sex	33F/45M (78)	20F/28M (48)	47F/36M (83)	0.069	0.099	0.944
Duration of illness, years^a^	1.4 ± 2.7 (78)	1.9 ± 3.4 (48)	-	-	-	0.333 (−1.0, 124)
BPRS positive symptoms score (congruent items PANSS & BPRS)	17.0 ± 6.4 (75)	13.2 ± 4.0 (46)	8.0 ± 0.4 (83)	<0.001 (12.1, 74)	<0.001 (8.5, 45)	<0.001 (4.1, 119)
BPRS negative symptoms score (congruent items PANSS & BPRS)	9.4 ± 3.8 (75)	9.2 ± 3.4 (46)	5.0 ± 0.2 (83)	<0.001 (10.0, 74)	<0.001 (8.5, 45)	0.813 (0.2, 119)
BPRS18 total symptoms score	38.4 ± 12.3 (75)	35.7 ± 10.0 (46)	18.6 ± 1.5 (83)	<0.001 (13.8, 76)	<0.001 (11.5, 46)	0.219 (1.2, 119)
Body mass index (kg/m^2^)	25.3 ± 5.1 (78)	25.1 ± 5.5 (48)	24.1 ± 4.1 (83)	0.109 (1.6, 159)	0.278 (1.1, 129)	0.796 (0.3, 124)
Estimated total intracranial volume (cm^3^)	1391.0 ± 232.0 (78)	1375.7 ± 205.9 (48)	1342.5 ± 202.0 (83)	0.162 (1.4, 159)	0.369 (0.9, 129)	0.717 (0.363, 124)
Cumulative exposure for antipsychotic medication^c^	20 628.0 ± 24 642.0 (78)	6712.0 ± 17 643.0 (47)	-	-	-	<0.001 (3.7, 119)

^a^Time since appearance of positive symptoms.

^c^Cumulative lifetime antipsychotic drug exposure up to the MR scanning day (in CPZ mg, based on CPZ equivalent conversion).

^*^Two-tailed *p*-value of a chi square test (sex), or t-test (all other variables).

#### Participants at one-year follow-up

At follow-up, the number of retained participants in each group were 34 FEPs, 23 CHRs, and 53 CTRs. We excluded two FEPs, one CHR and two CTRs with failed hippocampal segmentation, and two FEPs and one CHR with excess movement in the T1 sequence, in addition to the subjects who had dropped out from the study.

#### Clinical outcome trajectories

For analyses of clinical outcome trajectories, we divided patient groups to two groups based on follow-up evaluation on level of functioning, remission status and transition to psychosis.

The CHR group was divided into two groups based on follow-up time transition to psychosis (CHR converting (CHR-C *n* = 11) and CHR non-converting (CHR-NC *n* = 37)), resulting in a 23% psychosis transition rate in the CHR group during the one-year follow-up. Only seven out of these eleven CHR converting subjects were available for the analyses due to dropouts.

Level of functioning was measured using global assessment of functioning (GAF) that was binarized to poor (GAF < 65) or good performance (GAF ≥ 65) based on follow-up evaluation. Remission status (remission or non-remission) was defined using the scores of the Brief Psychiatric Rating Scale (BPRS) and three added items from Scale for the Assessment of Negative Symptoms (SANS) or the Positive and Negative Symptom Scale (SCI-PANSS) scores at the follow-up time point^46^. Sample sizes based on outcome measures are presented in Tables 5, 6.

### MRI acquisition and processing

**Fig. 1:**
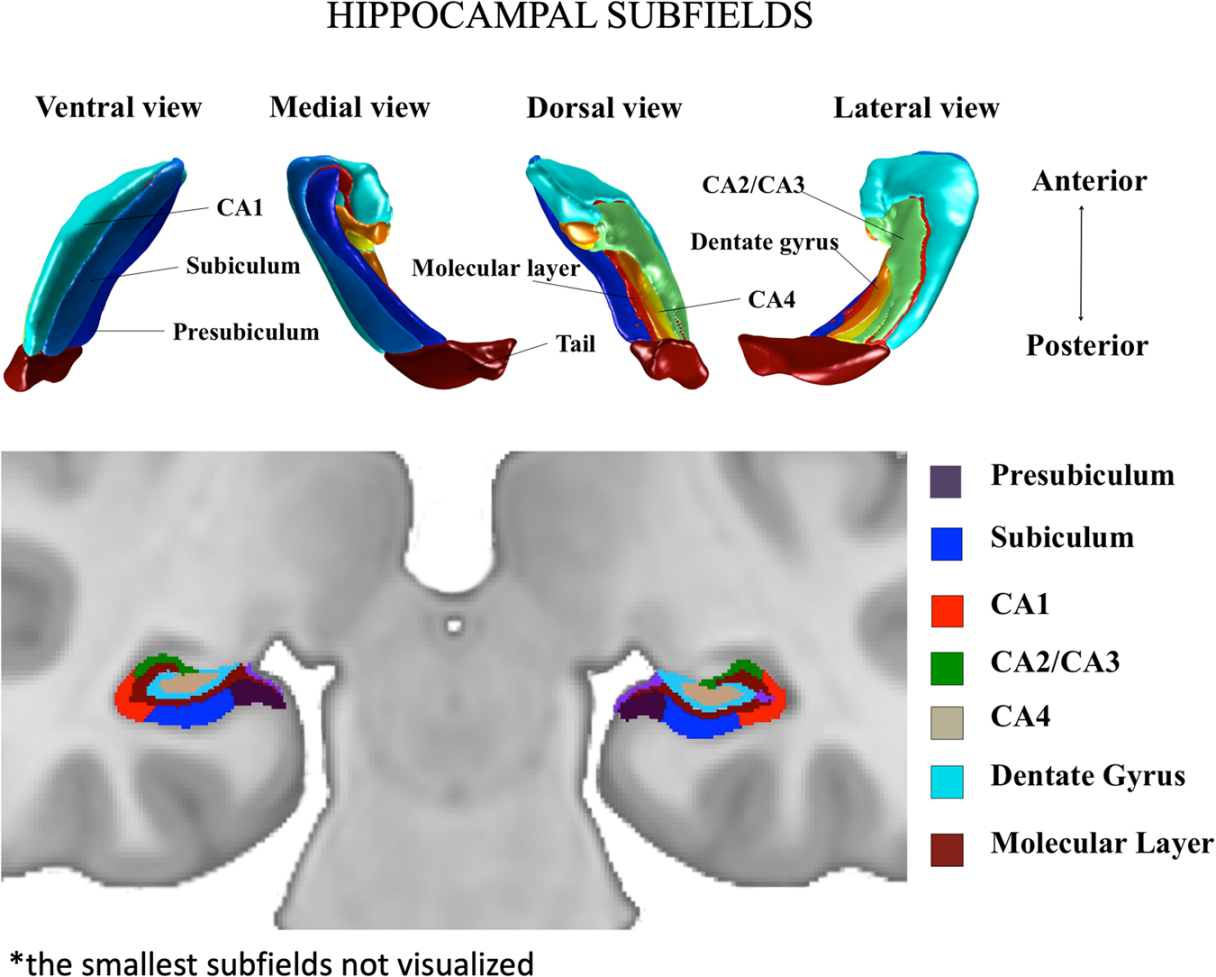
Visualization of hippocampal subfields. Upper: 3D-visualisation of hippocampal subfields generated with FreeSurfer. Lower: Hippocampal subfield segmentation masks shown overlaid on a representative coronal plane of T1-weighted MRI image.

The participants were scanned with a Philips Ingenuity TF 3-Tesla PET/MR scanner. A T1-weighted (Ultrafast Gradient Echo 3D, TR = 8.1 ms, TE-time = 3.7 ms, flip angle 7°, FOV = 256 x 256 x 176 mm^3^ and voxel size 1 x 1 x 1 mm^3^) image was acquired from all subjects. The T1 images were preprocessed, and the 12 hippocampal subfield volumes were segmented using the longitudinal FreeSurfer pipeline in version 7.1.1^47,48^. (https://surfer.nmr.mgh.harvard.edu/fswiki/HippocampalSubfieldsAndNucleiOfAmygdala). Quality control was done by visual inspection for all T1-weighted images and all segmented hippocampus in coronal, axial and sagittal planes by R-L A. The small head and body subdivisions were combined to correspond to each whole subfield and to reduce the number of comparisons. Eight subfields were chosen for statistical testing: the subiculum, cornu ammonis 1 (CA1), presubiculum, the molecular layer of the subiculum and the cornu ammonis -fields (ML), the granule cell layer and the molecular layer of the dentate gyrus (GCMLDG), cornu ammonis 2 and 3 (CA2 and CA3), cornu ammonis 4 (CA4) and hippocampal tail (Fig. 1). The hippocampal-amygdaloid transition region (HATA), hippocampal fissure and parasubiculum were excluded due to low reliability of segmenting regions with relatively small volumes compared to the voxel size.

### Clinical measures

Blood samples including fasting plasma glucose and fasting plasma insulin were acquired from the subjects (Table 2). The fasting glucose values included in the analysis were at non-diabetic level ranges (below 7.0 mmol/l; ranging from 4.0 mmol/l to 6.5 mmol/l). The homeostatic Model Assessment, HOMA2-IR (https://www.dtu.ox.ac.uk/homacalculator/) was used as a proxy for insulin resistance instead of HOMA1-IR^49,50^ since HOMA2-IR is considered to be a more accurate measure of insulin resistance and beta-cell function due to it representing both hepatic glucose output and peripheral glucose uptake.

**Table 2:** Glucose metabolism related parameters of the participants at the two timepoints.

	BASELINE						FOLLOW-UP					
	Mean ± SD (*n*)			*p*-value * (t-value, df)			Mean ± SD (n)			*p*-value * (t-value, df)		
	FEPs	CHRs	CTRs	FEP vs CTR	CHR vs CTR	FEP vs CHR	FEPs	CHRs	CTRs	FEP vs CTR	CHR vs CTR	FEP vs CHR
Fasting plasma glucose (mmol/l)	5.2 ± 0.4 (75)	5.3 ± 0.5 (38)	5.0 ± 0.4 (74)	0.002 (3.1, 147)	< 0.001 (3.7, 110)	0.155 (−1.4, 111)	5.3 ± 0.6 (29)	5.2 ± 0.6 (19)	5.1 ± 0.5 (49)	0.164 (1.4, 76)	0.700 (0.4, 66)	0.502 (0.7, 46)
Fasting plasma insulin (mU/l)	11.5 ± 8.1 (64)	10.6 ± 5.5 (33)	9.2 ± 5.2 (74)	0.048 (2.0, 105)	0.189 (1.3, 105)	0.572 (0.6, 95)	13.0 ± 10 (27)	18.0 ± 13.4 (17)	9.3 ± 4.8 (49)	0.076 (1.8, 33)	0.017 (2.6, 17)	0.158 (−1.4, 42)
Insulin resistance index (HOMA2-IR)	1.5 ± 1.0 (64)	1.4 ± 0.7 (33)	1.2 ± 0.7 (72)	0.062 (1.9, 106)	0.207 (1.3, 103)	0.620 (0.5, 95)	1.7 ± 1.2 (27)	2.3 ± 1.7 (17)	1.2 ± 0.6 (49)	0.073 (1.9, 34)	0.018 (2.6, 18)	0.161 (−1.4, 42)
Body mass index (kg/m^2^)	25.3 ± 5.1 (78)	25.1 /± 5.5 (48)	24.1 ± 4.1 (83)	0.109 (1.6, 159)	0.278 (1.1, 129)	0.796 (0.3, 124)	27.3 ± 6.6 (34)	26.8 ± 7.0 (23)	24.8 ± 4.3 (53)	0.058 (1.9, 51)	0.217 (1.3, 29)	0.786 (0.3, 55)

For fasting plasma glucose, insulin and HOMA2-IR see exclusions in the section ‘Clinical measures’.

*FEP* First-episode psychosis patient.

*CHR* Clinical high-risk patient.

*CTR* Population control.

*Two-tailed *p*-value of a t-test.

All antipsychotic daily doses preceding each scanning date were documented using available medical records. The daily doses were then converted to chlorpromazine (CPZ) equivalent daily dosages^51^ after which they were summed up to obtain the total cumulative lifetime antipsychotic exposures at each study time point. The information on the use of antidepressive and mood stabilizing medications were also collected (Supplementary table 3).

Symptom severity was assessed using either SCI-PANSS^52^ or BPRS (24- items, version 4.0). The PANSS scores were converted to correspond to the BPRS 18-item scores and the BPRS 24-item scores reduced to correspond to the BPRS 18-item scores^53^.

### Statistical analyses

#### Analyses of hippocampal volumetry and glucose parameters based on clinical groups of FEP, CHR and CTR

##### Between group differences of whole hippocampal and subfield volumes at baseline

Differences of whole hippocampus volumes between FEP, CHR and CTR were tested using a linear mixed-effects model with age, sex, body mass index (BMI) and scaled total intracranial volume (TIV) as covariates. Between-group comparisons of whole hippocampal volumes were done post-hoc using estimated marginal means using alpha levels corrected for false discovery rates.

Differences of hippocampal subfield volumes were tested between the three groups using a linear mixed-effects model: volume ~ intercept + β_1_ (group) + β_2_ (subfield) + β_3_ (group by subfield) + β_4_ (age) + β_5_ (sex) + β_6_ (TIV) + β_7_ (BMI) + random (subject) + ε. In the analysis, subfields were used as a within subject repeated measure. Volumes were deemed a dependent variable, while group status, subfield, interactions between group and subfield, age, sex, BMI, and TIV were the independent variables. Pairwise repeated measure between-group comparisons of subfield volumes were done post-hoc using estimated marginal means while correcting for false discovery rates.

##### Between group differences in parameters of glucose metabolism

Pairwise group differences of measured fasting plasma glucose and insulin, and calculated insulin resistance (HOMA2-IR) were tested using Student’s *t*-test in FEP, CHR and CTR both the baseline and follow-up time points. (Table 2)

##### Effect of fasting glucose, insulin and insulin resistance on hippocampal volumetry

Associations between parameters of glucose metabolism and volumes (subfield and total hippocampal volumes) were tested separately for FEP, CHR and CTR, for all metabolic indexes and for all volumes as well as in both time points using a linear model: volume ~ intercept + β_1_ (glucose parameter value) + β_2_ (age) + β_4_ (sex) + β_5_ (BMI) + β_6_ (total intracranial volume) + ε.

##### Linear mixed-effects model of longitudinal changes in subfield volumes and glucose metabolism parameters

We analyzed the baseline and one-year follow-up time points in the same linear mixed-effects model analysis to detect longitudinal changes in subfield volumes, total hippocampal volumes or glucose parameter values across time between FEP, CHR and CTR. These models included also subjects who did not have measurements at both time points. The linear mixed-effect models were performed separately for each volume and glucose parameter. Subject ID was used as a random effects variable. Post-hoc comparisons of volume or glucose parameter changes within each group between the baseline and one-year follow-up were performed using estimated marginal means.

The model included a binary independent variable that indicated the time point and its interaction with the group variable. The model used for the volume changes in time was: volume ~ intercept + β_1_ (group by time) + β_2_ (time) + β_3_ (group) + β_4_ (sex) + β_5_ (age) + β_6_ (total intracranial volume) + β_7_ (BMI) + random (subject) + ε. Model used for the glucose parameter value changes in time was: glucose parameter value ~ intercept + β_1_ (group by time) + β_2_ (time) + β_3_ (group) + β_4_ (sex) + β_5_ (age) + β_7_ (BMI) + random (subject) + ε. The post-hoc comparisons of volume or glucose parameter changes within each group between the baseline and one-year follow-up were performed using estimated marginal means.

#### Analyses of hippocampal volumetry and glucose parameters based on clinical outcomes

##### Transition to psychosis, remission and functioning during follow-up

A mixed-effects linear model was used to examine whether the reductions in subfield volumes at baseline, the baseline glucose parameter levels, or the longitudinal change in volumes or glucose parameters during the follow-up period, were associated with the clinical outcome trajectories, such as transition to psychosis, level of functioning or remission status at the follow-up.

With linear model, we also tested whether associations, between parameters of glucose metabolism and volumes, were based on clinical outcome trajectories.

#### Exploratory analyses

In the exploratory analyses we used linear mixed-effects model to detect differences in subfield or whole hippocampal volumes between non-affective psychosis and affective psychosis at baseline. Also, we explored possible differences in associations between hippocampal volumes and glucose parameters separately in NAP and AP at the two time points.

We collected the demographic information of the use of antidepressant and mood-stabilizing medications of the participants at the two time points. Due to the small sample size of the users of mood stabilizing medication, only linear model analyses of associations between lifetime antipsychotic exposure or the use of antidepressant medication and hippocampal total and subfield volumes were tested separately for studied clinical groups of FEP and CHR at the two time points. Further similar associations were studied in groups based on clinical outcome trajectories at the baseline.

The effect of lifetime antipsychotic exposure at each MRI scan time point was controlled by using it as a covariate for each test of this study separately. In all statistical analyses, two-tailed *p*-values < 0.05 were considered statistically significant. Baseline pairwise multiple comparisons of volumetric differences and analyses on associations between volumes and glucose parameters were corrected using false discovery rate (FDR) correction at *p*-value < 0.05.

## Results

### Analyses of hippocampal volumetry and glucose parameters based on clinical groups of FEP, CHR and CTR

#### Group differences of whole hippocampal and subfield volumes at baseline

There were no significant interactions between subfield volumes and hemispheres in single groups (hemisphere by subfield), nor were there interactions between the three groups (hemisphere by subfield by group). Consequently, all following tests were done using means of bilateral volumes as there was no hemispheric bias. All mean volumes were normally distributed (Shapiro Wilk’s test *p* > 0.05). There was no multicollinearity between the independent variables used in each model (Variance Inflation Factor, VIF < 2) (age, sex, TIV, BMI and fasting plasma glucose, fasting plasma insulin or insulin resistance).

The mean volume of the whole hippocampus was different between the three groups (F_2,202_ = 9.158, *p* < 0.001). Post-hoc pairwise tests showed that the mean whole hippocampus volume was significantly smaller in FEP compared to controls (estimated marginal means ± standard error: FEP = 3509 ± 38 mm^3^; CTR = 3645 ± 37 mm^3^), but not in FEP compared to CHR (CHR = 3603 ± 49 mm^3^). The difference between CHR and the control group was not significant.

The subfield volumes did not differ significantly between FEPs, CHRs and CTRs (subfield by group interaction F_14,1442_ = 1.33, *p* = 0.183). However, pairwise repeated measures analysis showed that in the FEP group, the volumes of the subiculum, presubiculum, molecular layer, CA1, GCMLDG and tail were significantly reduced compared to CTRs, whereas no statistically significant differences were observed in the combined CA2 and CA3 subfield or CA4 after FDR corrections (8 subfields and 3 contrasts per subfield; total 24 tests per FDR correction). FEPs had also significantly smaller CA1 volumes compared to CHRs, but no differences in other subfields were observed between FEP and CHR. Also, hippocampal tail was significantly smaller in CHR compared to CTR. (Table 3)

**Table 3:** a) Whole hippocampus volume pairwise between-group baseline comparison analysis of FEP, CHR and CTR, including age, sex, total intracranial volume and body mass index as covariates. b) Subregional post-hoc pairwise between-group baseline comparison analysis of FEP, CHR and CTR, including age, sex, total intracranial volume and BMI as covariates.

a) Volume	Contrast baseline	Estimated difference (mm^3^)	95% Confidence Interval	DF	t-ratio	*p*	FDR corrected p	p with exposure*	FDR corrected p with exposure*
Whole hippocampus	CTR - CHR	87	−15.09 –189	202	1.680	0.0944	0.0944	0.1306	0.1306
Whole hippocampus	CTR - FEP	192	103.50–281	202	4.274	<0.0001	<0.0001	0.0012	0.0035
Whole hippocampus	CHR - FEP	105	3.37–207	202	2.037	0.0429	0.0644	0.1137	0.1306

b) Volume	Contrast baseline	Estimated difference (mm^3^)	95% Confidence Interval	DF	t-ratio	*p*	FDR corrected p	p with exposure*	FDR corrected p with exposure*
Tail	CTR - CHR	27.28	10.40–44.2	202	3.187	0.0017	0.0100	0.0022	0.0130
Tail	CTR - FEP	34.44	19.78–49.1	202	4.632	<0.0001	0.0002	0.0001	0.0017
Tail	CHR - FEP	7.16	−9.77–24.1	202	0.834	0.4053	0.4229	0.5466	0.5704
Presubiculum	CTR - CHR	10.58	−6.29–27.5	202	1.236	0.2177	0.3486	0.2562	0.4645
Presubiculum	CTR - FEP	21.56	6.90–36.2	202	2.900	0.0041	0.0199	0.0188	0.0904
Presubiculum	CHR - FEP	10.98	−5.96–27.9	202	1.278	0.2026	0.3486	0.3142	0.4645
Subiculum	CTR - CHR	8.90	−7.97–25.8	202	1.040	0.2995	0.3626	0.3604	0.4645
Subiculum	CTR - FEP	18.88	4.22–33.5	202	2.539	0.0119	0.0444	0.0442	0.1404
Subiculum	CHR - FEP	9.97	−6.96–26.9	202	1.161	0.2469	0.3486	0.3593	0.4645
CA1	CTR - CHR	10.20	−6.68–27.1	202	1.192	0.2347	0.3486	0.3215	0.4645
CA1	CTR - FEP	31.44	16.78–46.1	202	4.229	<0.0001	0.0004	0.0005	0.0062
CA1	CHR - FEP	21.24	4.31–38.2	202	2.473	0.0142	0.0444	0.0296	0.1184
Molecular Layer	CTR - CHR	10.72	−6.16–27.6	202	1.252	0.2119	0.3486	0.2573	0.4645
Molecular Layer	CTR - FEP	29.98	15.32–44.6	202	4.032	0.0001	0.0006	0.0008	0.0062
Molecular Layer	CHR - FEP	19.26	2.33–36.2	202	2.243	0.0260	0.0624	0.0527	0.1404
GCMLDG	CTR - CHR	7.91	−8.96–24.8	202	0.925	0.3563	0.3887	0.3871	0.4645
GCMLDG	CTR - FEP	18.28	3.62–32.9	202	2.458	0.0148	0.0444	0.0523	0.1404
GCMLDG	CHR - FEP	10.36	−6.57–27.3	202	1.207	0.2290	0.2486	0.3678	0.4645
CA2/3	CTR - CHR	4.26	−12.62–21.1	202	0.498	0.6194	0.6194	0.6887	0.6887
CA2/3	CTR - FEP	13.64	−1.02–28.3	202	1.835	0.0680	0.1484	0.1812	0.3955
CA2/3	CHR - FEP	9.38	−7.55–26.3	202	1.092	0.2759	0.3626	0.4186	0.4784
CA4	CTR - CHR	8.32	−8.56–25.2	202	0.972	0.3321	0.3796	0.3664	0.4645
CA4	CTR - FEP	17.2	2.54–31.9	202	2.314	0.0217	0.0578	0.0707	0.1695
CA4	CHR - FEP	8.88	−8.05–25.8	202	1.034	0.3022	0.3626	0.4580	0.4997

*CA* Cornu ammonis, *Molecular Layer* Molecular Layer of the CA fields and subiculum, *GCMLDG* The Granule Cell and Molecular Layer of the Dentate Gyrus, *FDR* False discovery rate.

*Total lifetime antipsychotic exposure (until baseline) included in the model.

*FEP* First-episode psychosis patient.

*CHR* Clinical high-risk patient.

*CTR* Population control.

*Total lifetime antipsychotic exposure included in the model.

#### Between group differences in parameters of glucose metabolism

FEPs had significantly higher baseline fasting plasma and fasting insulin levels compared to CTR, while insulin resistance was higher only at a trend-level when compared to CTR. CHR had significantly higher fasting glucose values compared to CTR.

At the follow-up time point insulin and insulin resistance were higher in CHR while comparing to CTR. No such differences were seen between FEP and CHR at any time point. (Table 2)

#### Associations between parameters of glucose and hippocampal subfield volumetry

**Fig. 2:**
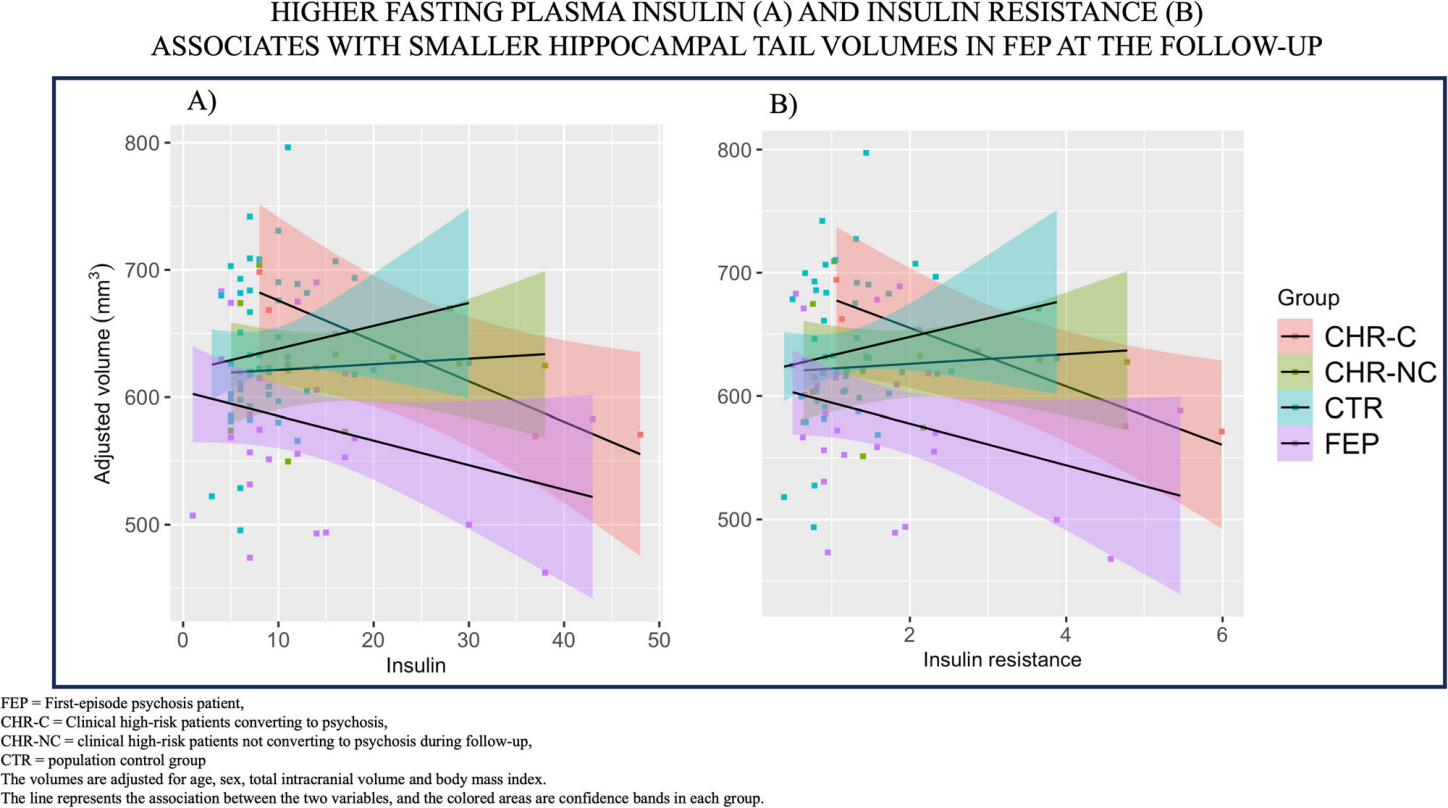
Associations between insulin or insulin resistance and hippocampal tail volumes. There is a significant subfield-specific inverse association between fasting plasma insulin (**A**) or insulin resistance (**B**) and hippocampal tail volumes in first-episode psychosis patients at the one-year follow-up time point. The model is adjusted for age, sex, TIV and BMI. Adjusting for lifetime antipsychotic exposure did not change these results.

There was a subfield specific effect of fasting insulin and insulin resistance in FEP at the follow-up time point (subfield by insulin F_3.4,72_ = 3.73, *p* = 0.011; subfield by insulin resistance F_3.4,71_ = 3.45, *p* = 0.017). We observed a significant inverse association between fasting plasma insulin or insulin resistance and hippocampal tail (insulin: t = −3.42, beta = −7.41, 95% CI [−11.9, −2.9], *p* = 0.003 FDR *p* = 0.024; insulin resistance: t = −17.85, beta = −58.90, 95% CI [−96.0, −21.8], *p* = 0.003, FDR *p* = 0.024) and between insulin resistance and molecular layer (t = −2.14, beta = −25.74, 95% CI [−50.8, −0.7], *p* = 0.044, FDR *p* = 0.186) in non-diabetic FEP patients, at the follow-up time point, but not at the baseline. After FDR correction for multiple comparisons (8 subfields and 2 glucose parameters; total 16 tests per FDR correction), only associations on hippocampal tail remained statistically significant (Fig. 2). Similar associations were not observed in CHR or CTR. Adjusting for lifetime exposure to antipsychotic medication, or the use of antidepressants did not change these results.

Also, there was a significant inverse association between insulin or insulin resistance and total hippocampal volume of non-diabetic FEPs at the follow-up (insulin: t = −2.43, beta = −21.31, 95% CI [−39.6, −3.0], p = 0.024; insulin resistance: t = −2.46, beta = −175.53, 95% CI [−323.8, −27.3], *p* = 0.023) (Supplementary fig. 1). Adjusting for lifetime exposure to antipsychotic medication or the use of antidepressants did not change these results. However, these results of total hippocampal volume did not survive the FDR correction (3 groups and 3 measures of glucose parameters; total 9 tests for FDR correction).

There were no significant associations between subfield volumes or total hippocampal volume and glucose parameters in any group at the baseline, or in CHR and CTR at the follow-up time point.

#### Longitudinal changes in subfield volumes and glucose metabolism parameters

There was a statistically significant increase in insulin (t_74_ = 3.334, *p* = 0.0013, estimate = 4.49, 95% CI [1.8, 7.2], ES (Cohen’s d) = 1.12, ES 95% CI [0.4, 1.8],) and insulin resistance (t_74_ = 3.232, *p* = 0.0018, estimate = 0.549, 95% CI [0.2, 0.9], ES (Cohen’s d) = 1.08, ES 95% CI [0.4,1.8]) levels in CHR, but not in FEP or CTR during the follow-up period.

When we compared the difference of the change of insulin and insulin resistance between groups, we found that the increase in fasting insulin and insulin resistance was significantly different in CHR compared to CTR (insulin: Group by time t_74_ = 3.119, p = 0.003; insulin resistance: Group by time t_74_ = 2.970, *p* = 0.004), and in CHR compared to FEP (insulin: Group by time t_74_ = 2.447, *p* = 0.016; Group by time t_74_ = 2.380, *p* = 0.020) but not in FEP compared to CTR. Adjusting for lifetime exposure to antipsychotic medication at in each time point did not change these results.

There were no changes in whole hippocampal or subfield volumes within the follow-up period in FEP, CHR or CTR in a longitudinal linear mixed-effects model.

### Analyses of hippocampal volumetry and glucose parameters based on clinical outcome groups

#### Baseline subfield volumetry and plasma glucose parameter levels based on outcome measures of transition, remission and functioning

**Fig. 3:**
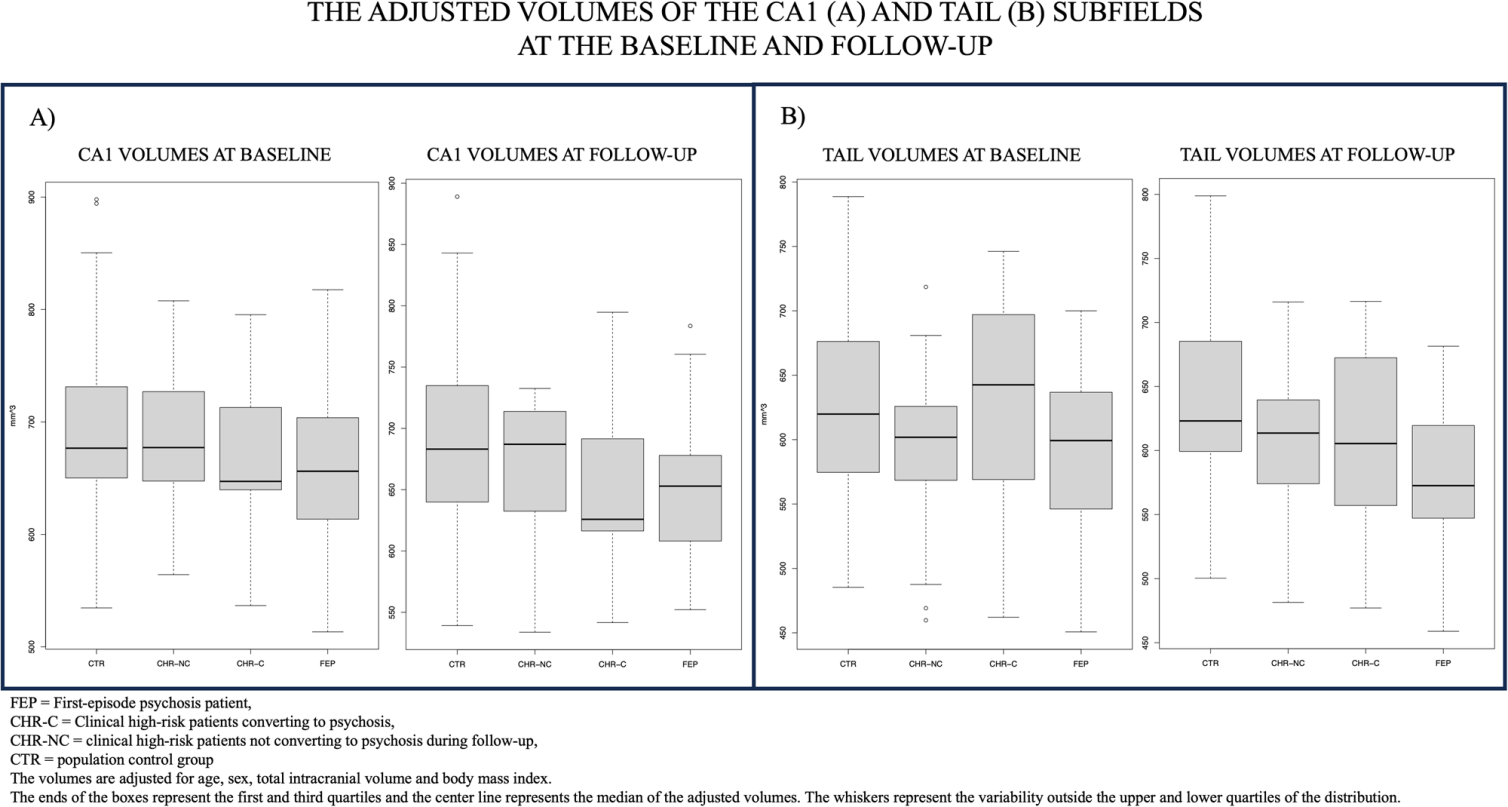
Volume differences of CA1 and tail subfields. Illustration of differences in CA1 and tail volumes of FEP, CHR-C, CHR-NC and CTR at baseline (**A**) and at one-year follow-up (**B**). The volumes are adjusted for age, sex, TIV and BMI.

The hippocampal tail volume was statistically significantly smaller in CHR-NC (*p* = 0.0002, FDR *p* = 0.0025), but not in CHR-C compared to CTR (*p* = 0.970, FDR *p* = 0.970). The difference in hippocampal tail volume between CHR-C and CHR-NC did not survive the FDR correction (*p* = 0.033, FDR *p* = 0.142). Also, the volumes of CA1 in the FEP group differed from those of CHR-NCs (*p* = 0.0099, FDR *p* = 0.079) but not from those of CHR-Cs (FDR *p* = 0.672). The tail volume was bigger in CHR-C compared to CHR-NC and CA1 was volume bigger in CHR-NC compared to FEP, but overall, these baseline differences did not survive FDR correction for multiple comparisons (Table 4 and Fig. 3). There were no differences between CHR-C and CHR-NC in whole hippocampal volumes at the baseline. The total hippocampal volume of CHR-NR, FEP-NR and FEP-R was smaller compared to CTR (Table 5). Comparisons of baseline volumes based on outcome measures are listed in Tables 4, 5 and 6.

**Table 4:** a) Whole hippocampus volume pairwise between-group baseline comparison analysis of FEP, CHR-C, CHR-NC and CTR, including age, sex, total intracranial volume and body mass index as covariates. b) Post-hoc pairwise between-group comparison analysis of FEP, CHR-C, CHR-NC and CTR at baseline, including age, sex, total intracranial volume and body mass index as covariates.

a) Volume	Contrast baseline	Estimated difference (mm^3^)	95% Confidence Interval	DF	t-ratio	*p*	FDR corrected p	p with exposure*	FDR corrected p with exposure*
Whole hippocampus	CTR – CHR-C	92	−87.05–272	201	1.015	0.3115	0.3738	0.4051	0.4861
Whole hippocampus	CTR – CHR-NC	85	−25.92–197	201	1.513	0.1318	0.2637	0.1596	0.3192
Whole hippocampus	CTR – FEP	192	103.29–281	201	4.264	<0.0001	0.0002	0.0012	0.0075
Whole hippocampus	CHR-C – CHR-NC	−7	−197.95–184	201	−0.070	0.9440	0.9440	0.9671	0.9671
Whole hippocampus	CHR-C – FEP	100	−78.99–279	201	1.101	0.2722	0.3738	0.3272	0.4861
Whole hippocampus	CHR-NC – FEP	107	−4.48–218	201	1.892	0.0599	0.1797	0.1559	0.3191

b) Volume	Contrast baseline	Estimated difference (mm^3^)	95% Confidence Interval	DF	t-ratio	*p*	FDR corrected p	p with exposure*	FDR corrected p with exposure*
Tail	CTR – CHR-C	0.568	−29.15–30.3	201	0.038	0.9700	0.969997	0.9801	0.98010
Tail	CTR – CHR-NC	35.217	16.83–53.6	201	3.777	0.0002	0.002507	0.0002	0.00586
Tail	CTR – FEP	34.438	19.77–49.1	201	4.630	<0.0001	0.000314	0.0001	0.00347
Tail	CHR-C – CHR-NC	34.650	2.92–66.4	201	2.153	0.0325	0.141819	0.0287	0.19129
Tail	CHR-C – FEP	33.870	4.15–63.6	201	2.247	0.0257	0.123527	0.0319	0.19129
Tail	CHR-NC – FEP	−0.779	−19.23–17.7	201	−0.083	0.9337	0.969997	0.7546	0.88019
Presubiculum	CTR – CHR-C	24.503	−5.21–54.2	201	1.626	0.1055	0.344815	0.1348	0.49764
Presubiculum	CTR – CHR-NC	6.445	−11.94–24.8	201	0.691	0.4902	0.672310	0.5319	0.78313
Presubiculum	CTR – FEP	21.561	6.89–36.2	201	2.899	0.0042	0.039946	0.0193	0.17844
Presubiculum	CHR-C – CHR-NC	−18.059	−49.79–13.7	201	−1.122	0.2631	0.657214	0.2983	0.74849
Presubiculum	CHR-C – FEP	−2.943	−32.67–26.8	201	−0.195	0.8454	0.922270	0.7898	0.88019
Presubiculum	CHR-NC – FEP	15.116	−3.33–33.6	201	1.616	0.1078	0.344815	0.1872	0.59909
Subiculum	CTR – CHR-C	15.371	−14.34–45.1	201	1.020	0.3090	0.657214	0.3697	0.76341
Subiculum	CTR – CHR-NC	6.981	−11.40–25.4	201	0.749	0.4549	0.661697	0.5127	0.78313
Subiculum	CTR – FEP	18.875	4.21–33.5	201	2.538	0.0119	0.081683	0.0452	0.24095
Subiculum	CHR-C – CHR-NC	−8.390	−40.12–23.3	201	−0.521	0.6027	0.781874	0.6457	0.81564
Subiculum	CHR-C – FEP	3.505	−26.22–33.2	201	0.232	0.8164	0.922270	0.8744	0.91240
Subiculum	CHR-NC – FEP	11.895	−6.55–30.3	201	1.271	0.2051	0.579074	0.3119	0.74849
CA1	CTR – CHR-C	20.706	−9.01–50.4	201	1.374	0.1710	0.512865	0.2272	0.68158
CA1	CTR – CHR-NC	7.077	−11.31–25.5	201	0.759	0.4487	0.661697	0.5526	0.78313
CA1	CTR – FEP	31.441	16.78–46.1	201	4.227	<0.0001	0.000861	0.0005	0.00867
CA1	CHR-C – CHR-NC	−13.629	−45.36–18.1	201	−0.847	0.3981	0.657214	0.4312	0.78313
CA1	CHR-C – FEP	10.735	−18.99–40.5	201	0.712	0.4772	0.672310	0.5260	0.78313
CA1	CHR-NC – FEP	24.364	5.91–42.8	201	2.604	0.0099	0.079216	0.0223	0.17844
Molecular Layer	CTR – CHR-C	12.422	−17.29–42.1	201	0.824	0.4108	0.657214	0.4824	0.78313
Molecular Layer	CTR – CHR-NC	10.212	−8.17–28.6	201	1.095	0.2747	0.657214	0.3114	0.74849
Molecular Layer	CTR – FEP	29.981	15.32–44.6	201	4.031	0.0001	0.001260	0.0008	0.00970
Molecular Layer	CHR-C – CHR-NC	−2.209	−33.94–29.5	201	−0.137	0.8909	0.950333	0.9461	0.96621
Molecular Layer	CHR-C – FEP	17.560	−12.16–47.3	201	1.165	0.2455	0.654537	0.2791	0.74849
Molecular Layer	CHR-NC – FEP	19.769	1.32–38.2	201	2.113	0.0358	0.143366	0.0732	0.29287
GCMLDG	CTR – CHR-C	5.454	−24.26–35.2	201	0.362	0.7178	0.865521	0.8029	0.88019
GCMLDG	CTR – CHR-NC	8.645	−9.74–27.0	201	0.927	0.3549	0.657214	0.3614	0.76341
GCMLDG	CTR – FEP	18.276	3.61–32.9	201	2.457	0.0148	0.089083	0.0534	0.25624
GCMLDG	CHR-C – CHR-NC	3.191	−28.54–34.9	201	0.198	0.8430	0.922270	0.7649	0.88019
GCMLDG	CHR-C – FEP	12.822	−16.90–42.5	201	0.851	0.3960	0.657214	0.4405	0.78313
GCMLDG	CHR-NC – FEP	9.631	−8.82–28.1	201	1.029	0.3045	0.657214	0.4831	0.78313
CA2/3	CTR – CHR-C	−0.756	−30.47–29.0	201	−0.050	0.9600	0.969997	0.8626	0.91240
CA2/3	CTR – CHR-NC	5.750	−12.64–24.1	201	0.617	0.5381	0.717524	0.5732	0.78313
CA2/3	CTR – FEP	13.640	−1.03–28.3	201	1.834	0.0681	0.251590	0.1840	0.59909
CA2/3	CHR-C – CHR-NC	6.506	−25.23–38.2	201	0.404	0.6864	0.865521	0.6234	0.80871
CA2/3	CHR-C – FEP	14.397	−15.33–44.1	201	0.955	0.3407	0.657214	0.3817	0.76341
CA2/3	CHR-NC – FEP	7.890	−10.56–26.3	201	0.843	0.4000	0.657214	0.5873	0.78313
CA4	CTR – CHR-C	5.384	−24.33–35.1	201	0.357	0.7213	0.865521	0.8068	0.88019
CA4	CTR – CHR-NC	9.194	−9.19–27.6	201	0.986	0.3253	0.657214	0.3365	0.76341
CA4	CTR – FEP	17.203	2.54–31.9	201	2.313	0.0217	0.115912	0.0721	0.29287
CA4	CHR-C – CHR-NC	3.810	−27.92–35.5	201	0.237	0.8131	0.922270	0.7397	0.88019
CA4	CHR-C – FEP	11.819	−17.90–41.5	201	0.784	0.4339	0.661697	0.4806	0.78313
CA4	CHR-NC – FEP	8.009	−10.44–26.5	201	0.856	0.3930	0.657214	0.5867	0.78313

*CA* Cornu ammonis, *Molecular Layer* Molecular Layer of the CA fields and subiculum, *GCMLDG* The Granule Cell and Molecular Layer of the Dentate Gyrus, *FDR* False discovery rate.

*FEP* First-episode psychosis, *CHR-C* Clinical high-risk patient converting to psychosis, *CHR-NC* CHR not converting to psychosis during the follow-up, *CTR* Population controls.

*Total lifetime antipsychotic exposure included in the model.

*Total lifetime antipsychotic exposure included in the model.

**Table 5:** a) Whole hippocampus volume pairwise between-group baseline comparison analysis of FEP-NR, FEP-R, CHR-NR, CHR-R and CTR, including age, sex, total intracranial volume and body mass index as covariates. b) Subregional post-hoc pairwise between-group baseline comparison analysis of FEP-NR, FEP-R, CHR-NR, CHR-R and CTR, including age, sex, total intracranial volume and BMI as covariates.

a) Volume	Contrast baseline	Estimated difference (mm^3^)	95 % Confidence Interval	DF	t-ratio	*p*	FDR corrected p	p with exposure*	FDR corrected p with exposure*
Whole hippocampus	CTR - CHR-NR	250	54.250–445.36	121	2.529	0.0127	0.0424	0.0243	0.0932
Whole hippocampus	CTR - CHR-R	28	−146.636–203.56	121	0.322	0.7482	0.8979	0.7569	0.8410
Whole hippocampus	CTR - FEP-NR	223	67.950–378.72	121	2.846	0.0052	0.0260	0.0278	0.0932
Whole hippocampus	CTR - FEP-R	215	67.189–363.37	121	2.878	0.0047	0.0260	0.0280	0.0932
Whole hippocampus	CHR-NR - CHR-R	−221	−451.942–9.25	121	−1.900	0.0598	0.1049	0.0892	0.2229
Whole hippocampus	CHR-NR - FEP-NR	−26	−241.754–188.81	121	−0.243	0.8081	0.8979	0.7089	0.8410
Whole hippocampus	CHR-NR - FEP-R	−35	−248.390–179.34	121	−0.320	0.7498	0.8979	0.6538	0.8410
Whole hippocampus	CHR-R - FEP-NR	195	−0.993–390.75	121	1.970	0.0512	0.1049	0.1228	0.2403
Whole hippocampus	CHR-R - FEP-R	187	−10.222–383.86	121	1.877	0.0629	0.1049	0.1442	0.2403
Whole hippocampus	FEP-NR - FEP-R	−8	−186.384–170.27	121	−0.089	0.9289	0.9289	0.9300	0.9300

b) Volume	Contrast baseline	Estimated difference (mm^3^)	95 % Confidence Interval	DF	t-ratio	*p*	FDR corrected p	p with exposure*	FDR corrected p with exposure*
Tail	CTR - CHR-NR	67.379	35.855–98.903	121	4.231	<0.0001	0.00182	0.0001	0.0072
Tail	CTR - CHR-R	19.973	−8.210–48.155	121	1.403	0.1632	0.38391	0.1656	0.5282
Tail	CTR - FEP-NR	43.389	18.690–68.089	121	3.478	0.0007	0.01875	0.0029	0.0771
Tail	CTR - FEP-R	51.407	27.511–75.303	121	4.259	<0.0001	0.00182	0.0003	0.0101
Tail	CHR-NR - CHR-R	−47.406	−84.796 to −10.017	121	−2.510	0.0134	0.15301	0.0187	0.3298
Tail	CHR-NR - FEP-NR	−23.989	−58.765–10.786	121	−1.366	0.1746	0.38792	0.1503	0.5282
Tail	CHR-NR - FEP-R	−15.972	−50.516–18.573	121	−0.915	0.3618	0.68920	0.3198	0.6560
Tail	CHR-R - FEP-NR	23.417	−8.270–55.104	121	1.463	0.1460	0.37689	0.2307	0.5558
Tail	CHR-R - FEP-R	31.434	−0.243–63.112	121	1.965	0.0518	0.27602	0.0927	0.4946
Tail	FEP-NR - FEP-R	8.018	−20.477–36.512	121	0.557	0.5785	0.82737	0.5779	0.8746
Presubiculum	CTR - CHR-NR	19.637	−11.887–51.162	121	1.233	0.2199	0.46288	0.2805	0.6066
Presubiculum	CTR - CHR-R	−6.195	−34.377–21.987	121	−0.435	0.6642	0.88558	0.6585	0.8746
Presubiculum	CTR - FEP-NR	20.706	−3.994–45.406	121	1.660	0.0996	0.31864	0.1958	0.5353
Presubiculum	CTR - FEP-R	25.921	2.025–49.817	121	2.148	0.0337	0.25340	0.0818	0.4946
Presubiculum	CHR-NR - CHR-R	−25.833	−63.222–11.557	121	−1.368	0.1739	0.38792	0.2141	0.5353
Presubiculum	CHR-NR - FEP-NR	1.069	−33.707–35.844	121	0.061	0.9516	0.97025	0.9787	0.9884
Presubiculum	CHR-NR - FEP-R	6.284	−28.261–40.828	121	0.360	0.7194	0.92645	0.7864	0.9399
Presubiculum	CHR-R - FEP-NR	26.901	−4.786–58.588	121	1.681	0.0954	0.31864	0.1591	0.5282
Presubiculum	CHR-R - FEP-R	32.116	0.439–63.794	121	2.007	0.0470	0.27484	0.0851	0.4946
Presubiculum	FEP-NR - FEP-R	5.215	−23.280–33.710	121	0.362	0.7177	0.92645	0.7170	0.9105
Subiculum	CTR - CHR-NR	9.147	−22.377–40.672	121	0.574	0.5667	0.82737	0.6669	0.8746
Subiculum	CTR - CHR-R	−1.670	−29.853–26.512	121	−0.117	0.9068	0.96723	0.9006	0.9510
Subiculum	CTR - FEP-NR	18.578	−6.122–43.278	121	1.489	0.1391	0.37086	0.2575	0.5723
Subiculum	CTR - FEP-R	21.118	−2.778–45.014	121	1.750	0.0827	0.31864	0.1717	0.5282
Subiculum	CHR-NR - CHR-R	−10.818	−48.207–26.572	121	−0.573	0.5678	0.82737	0.6472	0.8746
Subiculum	CHR-NR - FEP-NR	9.430	−25.345–44.206	121	0.537	0.5923	0.82737	0.6554	0.8746
Subiculum	CHR-NR - FEP-R	11.971	−22.574–46.515	121	0.686	0.4940	0.79039	0.5521	0.8746
Subiculum	CHR-R - FEP-NR	20.248	−11.439–51.935	121	1.265	0.2083	0.45032	0.3137	0.6560
Subiculum	CHR-R - FEP-R	22.789	−8.889–54.466	121	1.424	0.1570	0.38049	0.2452	0.5604
Subiculum	FEP-NR - FEP-R	2.540	−25.954–31.035	121	0.177	0.8602	0.96723	0.8594	0.9469
CA1	CTR - CHR-NR	38.794	7.270–70.318	121	2.436	0.0163	0.16294	0.0246	0.3298
CA1	CTR - CHR-R	0.749	−27.433–28.931	121	0.053	0.9581	0.97025	0.9644	0.9884
CA1	CTR - FEP-NR	32.708	8.008–57.408	121	2.622	0.0099	0.15301	0.0282	0.3298
CA1	CTR - FEP-R	28.220	4.324–52.116	121	2.338	0.0210	0.18692	0.0551	0.4408
CA1	CHR-NR - CHR-R	−38.045	−75.435 – −0.656	121	−2.014	0.0462	0.27484	0.0610	0.4433
CA1	CHR-NR - FEP-NR	−6.086	−40.862–28.689	121	−0.346	0.7296	0.92645	0.6661	0.8746
CA1	CHR-NR - FEP-R	−10.574	−45.118–23.971	121	−0.606	0.5457	0.82737	0.4910	0.8539
CA1	CHR-R - FEP-NR	31.959	0.272–63.646	121	1.997	0.0481	0.27484	0.0871	0.4946
CA1	CHR-R - FEP-R	27.471	−4.206–59.149	121	1.717	0.0886	0.31864	0.1488	0.5282
CA1	FEP-NR - FEP-R	−4.488	−32.982–24.007	121	−0.312	0.7557	0.94467	0.7565	0.9311
Molecular layer	CTR - CHR-NR	33.984	2.460–65.508	121	2.134	0.0348	0.25340	0.0504	0.4408
Molecular layer	CTR - CHR-R	2.531	−25.651–30.713	121	0.178	0.8592	0.96723	0.8653	0.9469
Molecular layer	CTR - FEP-NR	32.580	7.881–57.280	121	2.611	0.0102	0.15301	0.0289	0.3298
Molecular layer	CTR - FEP-R	30.816	6.920–54.712	121	2.553	0.0119	0.15301	0.0342	0.3419
Molecular layer	CHR-NR - CHR-R	−31.453	−68.842–5.937	121	−1.665	0.0984	0.31864	0.1251	0.5282
Molecular layer	CHR-NR - FEP-NR	−1.403	−36.179–33.372	121	−0.080	0.9365	0.97025	0.8677	0.9469
Molecular layer	CHR-NR - FEP-R	−3.168	−37.712–31.377	121	−0.182	0.8563	0.96723	0.7892	0.9399
Molecular layer	CHR-R - FEP-NR	30.050	−1.638–61.737	121	1.877	0.0629	0.31432	0.1103	0.5282
Molecular layer	CHR-R - FEP-R	28.285	−3.392–59.962	121	1.768	0.0796	0.31864	0.1355	0.5282
Molecular layer	FEP-NR - FEP-R	−1.764	−30.259–26.730	121	−0.123	0.9026	0.96723	0.9034	0.9510
GCMLDG	CTR - CHR-NR	22.724	−8.800–54.249	121	1.427	0.1561	0.38049	0.2044	0.5353
GCMLDG	CTR - CHR-R	7.342	−20.841–35.524	121	0.516	0.6070	0.82737	0.6125	0.8746
GCMLDG	CTR - FEP-NR	22.494	−2.206–47.193	121	1.803	0.0739	0.31864	0.1530	0.5282
GCMLDG	CTR - FEP-R	20.227	−3.669–44.123	121	1.676	0.0964	0.31864	0.1945	0.5353
GCMLDG	CHR-NR - CHR-R	−15.383	−52.772–22.007	121	−0.815	0.4170	0.73431	0.4859	0.8539
GCMLDG	CHR-NR - FEP-NR	−0.231	−35.006–34.545	121	−0.013	0.9895	0.98954	0.9201	0.9560
GCMLDG	CHR-NR - FEP-R	−2.498	−37.042–32.047	121	−0.143	0.8864	0.96723	0.8187	0.9469
GCMLDG	CHR-R - FEP-NR	15.152	−16.535–46.839	121	0.947	0.3457	0.67453	0.4845	0.8539
GCMLDG	CHR-R - FEP-R	12.885	−18.792–44.562	121	0.805	0.4222	0.73431	0.5736	0.8746
GCMLDG	FEP-NR - FEP-R	−2.267	−30.762–26.228	121	−0.157	0.8751	0.96723	0.8759	0.9469
CA2/3	CTR - CHR-NR	24.605	−6.919–56.129	121	1.545	0.1249	0.35685	0.1662	0.5282
CA2/3	CTR - CHR-R	7.670	−20.512–35.852	121	0.539	0.5910	0.82737	0.5964	0.8746
CA2/3	CTR - FEP-NR	20.066	−4.634–44.766	121	1.608	0.1104	0.33957	0.2130	0.5353
CA2/3	CTR - FEP-R	11.533	−12.363–35.429	121	0.956	0.3412	0.67453	0.5385	0.8746
CA2/3	CHR-NR - CHR-R	−16.935	−54.325–20.454	121	−0.897	0.3717	0.69144	0.4365	0.8516
CA2/3	CHR-NR - FEP-NR	−4.539	−39.314–30.236	121	−0.258	0.7965	0.95249	0.7309	0.9136
CA2/3	CHR-NR - FEP-R	−13.072	−47.617–21.473	121	−0.749	0.4552	0.75869	0.4063	0.8125
CA2/3	CHR-R - FEP-NR	12.396	−19.291–44.083	121	0.775	0.4401	0.74918	0.5947	0.8746
CA2/3	CHR-R - FEP-R	3.863	−27.814–35.541	121	0.241	0.8096	0.95249	0.9884	0.9884
CA2/3	FEP-NR - FEP-R	−8.533	−37.028–19.962	121	−0.593	0.5544	0.82737	0.5551	0.8746
CA4	CTR - CHR-NR	23.870	−7.654–55.394	121	1.499	0.1365	0.37086	0.1804	0.5346
CA4	CTR - CHR-R	7.276	−20.906–35.458	121	0.511	0.6102	0.82737	0.6157	0.8746
CA4	CTR - FEP-NR	22.474	−2.226–47.174	121	1.801	0.0741	0.31864	0.1534	0.5282
CA4	CTR - FEP-R	18.784	−5.112–42.681	121	1.556	0.1223	0.35685	0.2362	0.5558
CA4	CHR-NR - CHR-R	−16.594	−53.983–20.796	121	−0.879	0.3813	0.69334	0.4471	0.8516
CA4	CHR-NR - FEP-NR	−1.396	−36.171–33.380	121	−0.079	0.9368	0.97025	0.8680	0.9469
CA4	CHR-NR - FEP-R	−5.086	−39.630–29.459	121	−0.291	0.7712	0.94917	0.7065	0.9105
CA4	CHR-R - FEP-NR	15.198	−16.489–46.885	121	0.950	0.3442	0.67453	0.4828	0.8539
CA4	CHR-R - FEP-R	11.508	−20.169–43.186	121	0.719	0.4734	0.77286	0.6318	0.8746
CA4	FEP-NR - FEP-R	−3.690	−32.184–24.805	121	−0.256	0.7981	0.95249	0.7989	0.9399

*CA* cornu ammonis, *Molecular Layer* Molecular Layer of the CA fields and subiculum, *GCMLDG* The Granule Cell and Molecular Layer of the Dentate Gyrus, *FDR* false discovery rate.

*FEP* first-episode psychosis, *CHR-R* clinical high-risk patient in remission, *CHR-N* CHR not in remission, *FEP-R* first-episode psychosis patient in remission, *FEP-NR* FEP not in remission at the follow-up, *CTR* population controls.

*Total lifetime antipsychotic exposure included in the model.

Sample sizes:

FEP nonremission, FEP-NR: baseline *n* = 24, follow-up *n* = 12.

FEP remission, FEP-R: baseline *n* = 25, follow-up *n* = 22.

CHR nonremission, CHR-NR: baseline *n* = 12, follow-up *n* = 7.

CHR remission, CHR-R: baseline *n* = 16, follow-up *n* = 15.

CTR: baseline *n* = 53, follow-up *n* = 48.

*Total lifetime antipsychotic exposure included in the model.

**Table 6:** a) Whole hippocampus volume pairwise between-group baseline comparison analysis of FEP with good and poor GAF, CHR with good and poor GAF, and CTR, including age, sex, total intracranial volume and body mass index as covariates. b) Subregional post-hoc pairwise between-group baseline comparison analysis of FEP with good and poor GAF, CHR with good and poor GAF, and CTR, including age, sex, total intracranial volume and body mass index as covariates.

a) Volume	Contrast baseline	Estimated difference (mm^3^)	95 % Confidence Interval	DF	t-ratio	*p*	FDR corrected p	p with exposure*	FDR corrected p with exposure*
Whole hippocampus	CTR - CHR good GAF	69	−101.47–239	176	0.799	0.4256	0.5320	0.4565	0.5706
Whole hippocampus	CTR - CHR poor GAF	122	−6.14–249	176	1.879	0.0619	0.1950	0.0916	0.3053
Whole hippocampus	CTR - FEP good GAF	234	109.31–358	176	3.705	0.0003	0.0028	0.0025	0.0245
Whole hippocampus	CTR - FEP poor GAF	207	88.20–325	176	3.443	0.0007	0.0036	0.0054	0.0269
Whole hippocampus	CHR good GAF - CHR poor GAF	53	−130.89–236	176	0.566	0.5719	0.6354	0.6139	0.6821
Whole hippocampus	CHR good GAF - FEP good GAF	165	−18.66–348	176	1.773	0.0780	0.1950	0.1355	0.3387
Whole hippocampus	CHR good GAF - FEP poor GAF	138	−38.83–314	176	1.539	0.1255	0.2098	0.2044	0.3451
Whole hippocampus	CHR poor GAF - FEP good GAF	112	−31.76–256	176	1.538	0.1259	0.2098	0.2071	0.3451
Whole hippocampus	CHR poor GAF - FEP poor GAF	85	−46.03–216	176	1.280	0.2022	0.2888	0.3158	0.4511
Whole hippocampus	FEP good GAF - FEP poor GAF	−27	−162.83–109	176	−0.395	0.6934	0.6934	0.7046	0.7046

b) Volume	Contrast baseline	Estimated difference (mm^3^)	95 % Confidence Interval	DF	t-ratio	*p*	FDR corrected p	p with exposure*	FDR corrected p with exposure*
Tail	CTR - CHR good GAF	43.057	15.011–71.1	176	3.030	0.0028	0.032180	0.0032	0.05154
Tail	CTR - CHR poor GAF	31.901	11.129–52.7	176	3.031	0.0028	0.032180	0.0050	0.06716
Tail	CTR - FEP good GAF	44.942	24.461–65.4	176	4.331	<0.0001	0.000997	0.0001	0.00542
Tail	CTR - FEP poor GAF	42.572	23.479–61.7	176	4.401	<0.0001	0.000997	0.0001	0.00542
Tail	CHR good GAF - CHR poor GAF	−11.155	−41.328–19.0	176	−0.730	0.4666	0.673297	0.4304	0.72952
Tail	CHR good GAF - FEP good GAF	1.885	−28.281–32.1	176	0.123	0.9020	0.947032	0.9747	0.97584
Tail	CHR good GAF - FEP poor GAF	−0.485	−29.467–28.5	176	−0.033	0.9737	0.991538	0.8534	0.93283
Tail	CHR poor GAF - FEP good GAF	13.040	−10.443–36.5	176	1.096	0.2746	0.591672	0.3384	0.69426
Tail	CHR poor GAF - FEP poor GAF	10.670	−10.933–32.3	176	0.975	0.3310	0.608796	0.4024	0.72952
Tail	FEP good GAF - FEP poor GAF	−2.370	−24.383–19.6	176	-0.212	0.8320	0.941016	0.8398	0.93283
Presubiculum	CTR - CHR good GAF	2.382	−25.664–30.4	176	0.168	0.8671	0.947032	0.8933	0.93283
Presubiculum	CTR - CHR poor GAF	13.210	−7.562–34.0	176	1.255	0.2111	0.496743	0.2545	0.62526
Presubiculum	CTR - FEP good GAF	27.346	6.865–47.8	176	2.635	0.0092	0.081442	0.0243	0.21628
Presubiculum	CTR - FEP poor GAF	18.261	−0.831–37.4	176	1.888	0.0607	0.242904	0.1253	0.45572
Presubiculum	CHR good GAF - CHR poor GAF	10.828	−19.345–41.0	176	0.708	0.4797	0.673297	0.5039	0.72952
Presubiculum	CHR good GAF - FEP good GAF	24.965	−5.201–55.1	176	1.633	0.1042	0.347342	0.1471	0.49030
Presubiculum	CHR good GAF - FEP poor GAF	15.879	−13.103–44.9	176	1.081	0.2810	0.591672	0.3623	0.72465
Presubiculum	CHR poor GAF - FEP good GAF	14.136	−9.347–37.6	176	1.188	0.2364	0.540404	0.3153	0.68165
Presubiculum	CHR poor GAF - FEP poor GAF	5.051	−16.552–26.7	176	0.461	0.6451	0.781889	0.7702	0.89295
Presubiculum	FEP good GAF - FEP poor GAF	−9.085	−31.098–12.9	176	-0.815	0.4165	0.640706	0.4232	0.72952
Subiculum	CTR - CHR good GAF	−6.238	−34.284–21.8	176	−0.439	0.6612	0.789540	0.6387	0.82094
Subiculum	CTR - CHR poor GAF	14.037	−6.735–34.8	176	1.334	0.1840	0.466288	0.2313	0.61681
Subiculum	CTR - FEP good GAF	25.544	5.064–46.0	176	2.461	0.0148	0.118410	0.0368	0.26736
Subiculum	CTR - FEP poor GAF	14.154	−4.939–33.2	176	1.463	0.1452	0.446912	0.2589	0.62526
Subiculum	CHR good GAF - CHR poor GAF	20.275	−9.898–50.4	176	1.326	0.1865	0.466288	0.2060	0.61681
Subiculum	CHR good GAF - FEP good GAF	31.782	1.616–61.9	176	2.079	0.0390	0.209464	0.0596	0.36140
Subiculum	CHR good GAF - FEP poor GAF	20.391	−8.591–49.4	176	1.389	0.1667	0.454222	0.2256	0.61681
Subiculum	CHR poor GAF - FEP good GAF	11.507	−11.976–35.0	176	0.967	0.3348	0.608796	0.4201	0.72952
Subiculum	CHR poor GAF - FEP poor GAF	0.116	−21.487–21.7	176	0.011	0.9915	0.991538	0.8978	0.93283
Subiculum	FEP good GAF - FEP poor GAF	−11.391	−33.404–10.6	176	−1.021	0.3086	0.608796	0.3144	0.68165
CA1	CTR - CHR good GAF	2.962	−25.084–31.0	176	0.208	0.8352	0.941016	0.8613	0.93283
CA1	CTR - CHR poor GAF	14.491	−6.281–35.3	176	1.377	0.1703	0.454222	0.2200	0.61681
CA1	CTR - FEP good GAF	38.404	17.923–58.9	176	3.701	0.0003	0.007665	0.0012	0.03160
CA1	CTR - FEP poor GAF	28.451	9.359–47.5	176	2.941	0.0037	0.037125	0.0116	0.11591
CA1	CHR good GAF - CHR poor GAF	11.529	−18.644–41.7	176	0.754	0.4518	0.669341	0.4892	0.72952
CA1	CHR good GAF - FEP good GAF	35.442	5.276–65.6	176	2.319	0.0216	0.156794	0.0344	0.26736
CA1	CHR good GAF - FEP poor GAF	25.489	−3.493–54.5	176	1.736	0.0844	0.311262	0.1210	0.45572
CA1	CHR poor GAF - FEP good GAF	23.913	0.430–47.4	176	2.010	0.0460	0.216441	0.0678	0.36140
CA1	CHR poor GAF - FEP poor GAF	13.960	−7.643–35.6	176	1.275	0.2039	0.494247	0.2657	0.62526
CA1	FEP good GAF - FEP poor GAF	−9.953	−31.966–12.1	176	-0.892	0.3735	0.613373	0.3799	0.72952
Molecular layer	CTR - CHR good GAF	5.509	−22.537–33.6	176	0.388	0.6988	0.810155	0.7241	0.86455
Molecular layer	CTR - CHR poor GAF	14.986	−5.786–35.8	176	1.424	0.1563	0.454222	0.1963	0.61681
Molecular layer	CTR - FEP good GAF	35.557	15.076–56.0	176	3.426	0.0008	0.015234	0.0028	0.05154
Molecular layer	CTR - FEP poor GAF	30.312	11.219–49.4	176	3.133	0.0020	0.032180	0.0069	0.07844
Molecular layer	CHR good GAF - CHR poor GAF	9.477	−20.696–39.7	176	0.620	0.5361	0.711670	0.5668	0.76857
Molecular layer	CHR good GAF - FEP good GAF	30.048	−0.118–60.2	176	1.966	0.0509	0.226177	0.0761	0.38069
Molecular layer	CHR good GAF - FEP poor GAF	24.803	−4.179–53.8	176	1.689	0.0930	0.323476	0.1323	0.46006
Molecular layer	CHR poor GAF - FEP good GAF	20.571	−2.912–44.1	176	1.729	0.0856	0.311262	0.1239	0.45572
Molecular layer	CHR poor GAF - FEP poor GAF	15.326	−6.277–36.9	176	1.400	0.1632	0.454222	0.2244	0.61681
Molecular layer	FEP good GAF - FEP poor GAF	−5.245	−27.258–16.8	176	-0.470	0.6388	0.781889	0.6465	0.82094
GCMLDG	CTR - CHR good GAF	8.669	−19.377–36.7	176	0.610	0.5426	0.711670	0.5659	0.76857
GCMLDG	CTR - CHR poor GAF	10.846	−9.926–31.6	176	1.030	0.3042	0.608796	0.3379	0.69426
GCMLDG	CTR - FEP good GAF	21.270	0.789–41.8	176	2.050	0.0419	0.209464	0.0894	0.39721
GCMLDG	CTR - FEP poor GAF	21.503	2.410–40.6	176	2.223	0.0275	0.169304	0.0643	0.36140
GCMLDG	CHR good GAF - CHR poor GAF	2.177	−27.996–32.4	176	0.142	0.8869	0.947032	0.8926	0.93283
GCMLDG	CHR good GAF - FEP good GAF	12.601	−17.565–42.8	176	0.824	0.4108	0.640706	0.5107	0.72952
GCMLDG	CHR good GAF - FEP poor GAF	12.834	−16.149–41.8	176	0.874	0.3834	0.613373	0.4793	0.72952
GCMLDG	CHR poor GAF - FEP good GAF	10.424	−13.059–33.9	176	0.876	0.3822	0.613373	0.5048	0.72952
GCMLDG	CHR poor GAF - FEP poor GAF	10.657	−10.946–32.3	176	0.974	0.3316	0.608796	0.4512	0.72952
GCMLDG	FEP good GAF - FEP poor GAF	0.233	−21.780–22.2	176	0.021	0.9834	0.991538	0.9758	0.97584
CA2/3	CTR - CHR good GAF	6.578	−21.467–34.6	176	0.463	0.6440	0.781889	0.6687	0.82234
CA2/3	CTR - CHR poor GAF	9.365	−11.407–30.1	176	0.890	0.3748	0.613373	0.4232	0.72952
CA2/3	CTR - FEP good GAF	15.503	−4.978–36.0	176	1.494	0.1370	0.438400	0.2422	0.62495
CA2/3	CTR - FEP poor GAF	20.042	0.949–39.1	176	2.072	0.0398	0.209464	0.0878	0.39721
CA2/3	CHR good GAF - CHR poor GAF	2.787	−27.386–33.0	176	0.182	0.8556	0.947032	0.8722	0.93283
CA2/3	CHR good GAF - FEP good GAF	8.925	−21.241–39.1	176	0.584	0.5601	0.722647	0.6735	0.82234
CA2/3	CHR good GAF - FEP poor GAF	13.463	−15.519–42.4	176	0.917	0.3605	0.613373	0.4536	0.72952
CA2/3	CHR poor GAF - FEP good GAF	6.138	−17.345–29.6	176	0.516	0.6066	0.770314	0.7390	0.86939
CA2/3	CHR poor GAF - FEP poor GAF	10.677	−10.926–32.3	176	0.975	0.3307	0.608796	0.4390	0.72952
CA2/3	FEP good GAF - FEP poor GAF	4.539	−17.474–26.6	176	0.407	0.6846	0.805363	0.6784	0.82234
CA4	CTR - CHR good GAF	10.081	−17.965–38.1	176	0.709	0.4790	0.673297	0.5010	0.72952
CA4	CTR - CHR poor GAF	11.782	−8.990–32.6	176	1.119	0.2645	0.587766	0.3001	0.68165
CA4	CTR - FEP good GAF	20.009	−0.472–40.5	176	1.928	0.0555	0.233515	0.1133	0.45572
CA4	CTR - FEP poor GAF	21.567	2.475–40.7	176	2.229	0.0271	0.169304	0.0634	0.36140
CA4	CHR good GAF - CHR poor GAF	1.701	−28.472–31.9	176	0.111	0.9115	0.947032	0.9220	0.94561
CA4	CHR good GAF - FEP good GAF	9.928	−20.238–40.1	176	0.650	0.5168	0.700810	0.6270	0.82094
CA4	CHR good GAF - FEP poor GAF	11.487	−17.496–40.5	176	0.782	0.4352	0.656836	0.5370	0.75375
CA4	CHR poor GAF - FEP good GAF	8.227	−15.256–31.7	176	0.691	0.4902	0.676192	0.6206	0.82094
CA4	CHR poor GAF - FEP poor GAF	9.786	−11.818–31.4	176	0.894	0.3726	0.613373	0.4936	0.72952
CA4	FEP good GAF - FEP poor GAF	1.559	−20.454–23.6	176	0.140	0.8890	0.947032	0.8818	0.93283

*CA* cornu ammonis, *Molecular Layer* Molecular Layer of the CA fields and subiculum, *GCMLDG* The Granule Cell and Molecular Layer of the Dentate Gyrus, *FDR* False discovery rate.

*FEP* First-episode psychosis, *CHR* Clinical high-risk patient, *CTR* population controls, *GAF* Global assessment of functioning.

*Total lifetime antipsychotic exposure included in the model.

Sample sizes:

FEP poor GAF: baseline *n* = 45, follow-up *n* = 15.

FEP good GAF: baseline *n* = 33, follow-up *n* = 19.

CHR poor GAF: baseline *n* = 33, follow-up *n* = 16.

CHR good GAF: baseline *n* = 14, follow-up *n* = 7.

CTR: baseline *n* = 60, follow-up *n* = 53.

*Total lifetime antipsychotic exposure included in the model.

There were no significant differences in baseline glucose parameter measures between CHR-C and CHR-NC, between FEPs with poor and good GAF or between CHRs with poor and good GAF. Further, no differences in the glucose parameters between nonremitting and remitting FEPs or nonremitting and remitting CHRs.

#### Associations between hippocampal volumetry and glucose parameters based on clinical outcomes

There was a trend of negative association between fasting plasma insulin or insulin resistance and hippocampal tail, but also between fasting plasma insulin or insulin resistance and total hippocampal volume at the follow-up time point of CHR converted to psychosis (Fig. 2 and Supplementary figure 1). However, the group of CHR-C was too small to study associations between glucose parameters and volumetry at the two time points. No associations between glucose parameters and hippocampal subfields or total volumes were observed in the subgroups of remission and functioning of FEP or CHR at either time point.

#### Longitudinal changes in glucose parameters and hippocampal volumetry based on outcome trajectories

**Fig. 4:**
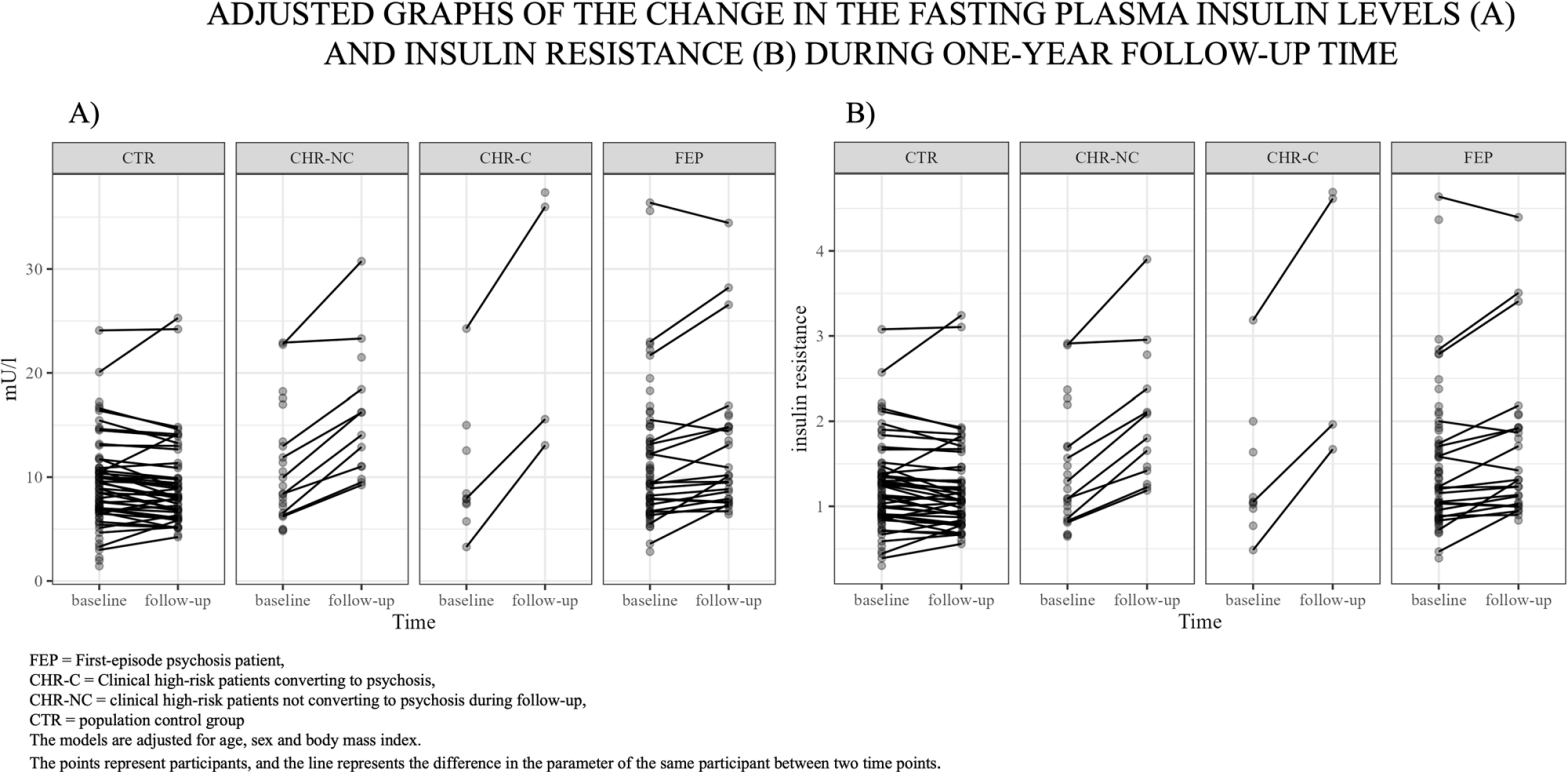
Longitudinal changes in insulin and insulin resistance. The change of fasting plasma insulin (**A**) and insulin resistance (**B**) during one-year follow-up. The increase in fasting plasma insulin and insulin resistance is statistically significant only in CHR-C. The model is adjusted for age, sex, and BMI. Adjusting for lifetime antipsychotic exposure did not change these results.

During the follow-up period, insulin (t_73_ = 3.705, p = 0.0004, estimate = 9.97, 95% CI [4.6, 15.3], ES (Cohen’s d) = 2.5, ES 95% CI [1.1, 3.9],) and insulin resistance (t_73_ = 3.539, *p* = 0.0007, estimate = 1.205, 95% CI [0.5, 1.9], ES (Cohen’s d) = 2.4, ES 95% CI [1.0, 3.8],) levels significantly increased in CHR-C, but not in CHR-NC (Fig. 4). This result was not affected by adjusting the model for lifetime antipsychotic exposure. The longitudinal change in insulin and insulin resistance levels of CHR-C differed significantly from the change of CTRs (insulin: group by time t_73_ = 3.684, *p* < 0.001 and insulin resistance: group by time t_73_ = 3.501, *p* < 0.001). Similar differences were not observed between CHR-NCs and CTRs or FEPs and CTRs.

Also, insulin and insulin resistance increased significantly in CHR-NRs (insulin: t_68_ = 2.886, *p* = 0.0052, estimate = 7.513, 95% CI [2.3, 12.7], ES (Cohen’s d) = 1.9, ES 95% CI [0.5, 3.2]; insulin resistance: t_68_ = 2.849, *p* = 0.0058, estimate = 0.9321, 95% CI [0.3, 1.6], ES (Cohen’s d) = 1.8, ES 95% CI [0.5, 3.1]) and in CHRs with poor GAF (insulin: t_72_ = 3.573, p = 0.0006, estimate = 5.784, 95% CI [2.6, 9.0], ES (Cohen’s d) = 1.4, ES 95% CI [0.6, 2.2]); insulin resistance: t_72_ = 3.477, *p* = 0.0009, estimate = 0.7092, 95% CI [0.3, 1.1], ES (Cohen’s d) = 1.4, ES 95% CI [0.6, 2.2]) during the follow-up period. This was not observed in other groups. Adjusting for lifetime antipsychotic exposure did not change these results.

Further, the volume of the presubiculum in FEPs with good GAF increased significantly during the follow-up period (presubiculum: t_91_ = 3.079 p = 0.0027, estimate = 5.314, 95% CI [1.9, 8.7], ES (Cohen’s d) = 1.2, ES 95% CI [0.4, 1.9]). This result remained significant after adjusting for lifetime antipsychotic exposure. There were no significant longitudinal changes in subfield or total hippocampal volumes in CHR-C, CHR-NC, nonremitters, remitters or patients with poor or good GAF in FEP or CHR during the follow-up period.

### Exploratory analyses

Non-affective psychosis had significantly smaller hippocampal tail volume compared to CTR (estimate = 42.05, 95% CI [25.9, 58.2] t_201_ = 5.14 *p* < 0.0001, FDR *p* < 0.0001). Similar difference was not observed between affective psychosis and CTR in the exploratory analyses using linear mixed-effects model. The hippocampal tail volume was significantly smaller in NAP compared to AP, but it did not survive FDR correction for multiple comparisons. There were no differences in other subfields or whole hippocampal volumes between NAP and AP. However, it seemed that there were more widespread reductions in the hippocampus in NAP compared to CTR than AP compared to CTR (Supplementary Table 4).

There were no significant associations between hippocampal total volume and glucose parameters, or subregion specific effects of glucose parameters on subfields in NAP or AP at the two time points. There was no significant difference in associations between fasting insulin or insulin resistance and hippocampal tail volume between the groups of NAP and AP at the follow-up.

The demographic information of the use of antidepressant and mood stabilizing medication at the two time points, and the results of the exploratory analyses of associations between lifetime antipsychotic exposure or antidepressant usage and hippocampal volumes using linear model, are presented in Supplementary table 3.

## Discussion

Our study supports the view that hippocampal subfield morphology deficits are partly developmental and stable near the onset of first psychosis, being more robust in non-affective psychoses, but less prominent in the CHR group, that includes both patients transitioning and not transitioning to psychosis. This highlights the temporospatial dimension of hippocampal abnormalities in the development of psychotic disorders. A novel finding in this study is the significant inverse association between fasting plasma insulin or insulin resistance and hippocampal tail in non-diabetic FEP patients, at the one-year follow-up time point, independent of antipsychotic and antidepressant medications. Also, we found that insulin and insulin resistance worsened in CHR during the one-year follow-up period. In CHR, the worsening was related to clinical outcome trajectories such as transition to psychosis, non-remission and poor level of functioning. Similar longitudinal findings of the changes in glucose metabolism were not observed in CTR, FEP, non-converting CHR, or related to clinical outcome in FEP.

Our MRI imaging analysis of hippocampus subfields in early psychosis are mainly in line with earlier studies^20,23,54^. There was an overall reduction of subfield hippocampus volumes in patients with first-episode psychosis compared to population controls. This volume defect stayed stable during the one-year follow-up. The most pronounced volume reductions in the FEP group were observed in tail, CA1 and molecular layer of CA fields, independent of lifetime antipsychotic exposure. A similar trend of reduction in CA1 was also observed in subgroup of CHRs converting to psychosis. Instead, in CHR, only the hippocampal tail was smaller compared to CTRs, especially in those not converting to psychosis. This might mean that volume reduction in tail of CHR is not specific to psychosis or it might be related to the effect of antidepressants^55,56^ However, there was no association between the use of antidepressants and tail volume in the patient groups. Also, it is possible that some CHR-NC transition to psychosis only after the one-year follow-up period. Thus, these glucometabolic and subfield-specific findings in CHR converting to psychosis, and those in FEP that relate to glucose metabolism, may provide additional temporospatial information that represents traits and states differently in the progression of psychotic syndrome.

### Temporospatial subfield defects are stable in early psychosis

The hippocampus is considered a key hub region in schizophrenia pathophysiology. The observed changes in hippocampus morphology are affected by the interaction of genes and the environment (GxE). The total volume of the hippocampus is moderately heritable in schizophrenia^57^ but there may be differences in the developmental trajectories of the separate hippocampal subfields^58^ and between posterior and anterior parts, but also between head-body-tail-division of the hippocampus^59,60^. These different divisions are partly overlapping which complicates the comparison and interpretation of the earlier studies on hippocampal morphology and related phenomena. Heritability of volume seems to be highest in the molecular layer, CA1, tail and DG, whereas smaller volume subfields, which were excluded from these analyses, seem to be less influenced by genetic factors in healthy twin studies^61^. Additionally, the reduced volume of the composite anterior CA subfields appears as a manifestation of the genetic vulnerability to schizophrenia also in unaffected relatives of schizophrenia patients^62^. A greater impact of genetic factors on the volumes of CA1 and molecular layer of CA fields is in line with our findings of a stable and possibly a developmentally early reduction of hippocampal subfield volumes. There is also evidence of hippocampal tail volume showing enrichment for schizophrenia-related genes^58^. In our sample, the majority of CHR-C converted to non-affective psychosis, and the majority of FEPs had a diagnosis of non-affective psychosis. Thus, these groups can be interpreted to primarily represent schizophrenia-spectrum psychoses, that have been shown to inherit a genetic liability to relatively smaller hippocampal volumes^63,64^. However, it is possible that the CA1 deficits are even more specific for schizophrenia^22,54^. Volume changes in the anterior hippocampus^15^, the region that is dominated by CA1 and subiculum subfield volumes^65^ have also been previously reported in genetic^58,66^ and MRI studies^54,67,68^ of psychoses. However, the relation between the anterior parts of hippocampus and schizophrenia seems to be more genetically driven, whereas the global reduction of hippocampal volume seen in later disease stages might be more strongly influenced by environmental factors and the disease process^58^.

Hypofunction of the N-methyl-D-aspartate (NMDA) glutamate receptor, as well as glutamate itself, are proposed to play a key role in the pathophysiology of schizophrenia^69^. There is evidence that the NMDA receptor, particularly in the CA1 subfield, has specific role in the function of the hippocampal comparator system involved in the processing of the match-mismatch of neural activity^70^, as well as in behavioral inhibition^71^. In addition, there is evidence of glutamate as a driver of hypermetabolism and atrophy starting in CA1 and spreading to the subiculum^8,67^. The CA1 subfield seems to be involved already in the prodromal phase of psychosis as implicated by elevated cerebral blood flow, hypermetabolism and volume loss in clinical high-risk patients who later convert to psychosis^23,72,73^ These findings further indicate that the changes in CA1 volumes seen in psychotic disorders are developmental in nature. In addition, the functions usually linked to the anterior region of the hippocampus such as regulation of anxiety and stress, sense of novelty, encoding, decision making, perception, imagination and episodic memory^65^, are all dimensions highly relevant for the clinical phenotypes of a patient with psychotic disorder.

Also, it has been shown that hippocampal function is altered in psychotic states with early life stress^74,75^. This might be due to abnormal input from the basolateral amygdala^76^. Volume reductions of the lateral nucleus of the amygdala are also related to childhood adversities in FEP^77^. The hippocampus is involved in the regulation of stress responses^78^, and stress on the other hand is associated with altered glucose metabolism^79,80^. Stress has also been suggested to induce changes in the morphology and dendrite spine density of the subiculum, CA1, CA3 and dentate gyrus^76,78,81–83^. Out of these regions, the CA1 and CA3 seem to be the primary hippocampal subfields influencing stress responses, including HPA axis activity^78,84^. On the other hand, hyperglycemia induces changes in synaptic function and neuronal loss particularly in the CA1 and CA3 subfields, in the studies on diabetic rats^27,29,85,86^. Hippocampal tail was not studied in these publications. Thus, the possible developmental defects in these subregions, especially in CA1, might be related to the development of dysregulation of glucose metabolism in patients who develop psychosis.

### Glucose metabolism near the onset of psychosis

In this study, we found that insulin resistance worsened during the prodromal period near the onset of first psychosis. This dysregulation of glucose metabolism may be explained by genetic factors but also by the combined effect of non-specific factors such as insomnia, poor nutrition and stress. However, previous studies^87,88^ support our results that the changes in glucose homeostasis seem to increase during the prodrome and intensify when the onset of the first psychotic episode draws closer. Since we found worsening of insulin and insulin resistance in CHR, most of whom do not develop psychosis, it would support the view that worsening of glucose metabolism is more commonly related to non-psychotic mental illness progression^40^. However, the worsening of glucose metabolism was statistically significant only in CHR-C, but not in CHR-NC. Also, the volumetric association with insulin and insulin resistance was observed only in FEP, which suggests a different mechanism in relation to psychotic illness.

Previous studies have shown that insulin signaling, and glucose homeostasis are dysregulated in first-episode psychosis independent of antipsychotic treatment^11,35,89,90^. This is in line with the results of this study: FEPs had the most severe deviation of glucose parameters compared to CHRs and CTRs. Further, there is evidence suggesting that insulin signaling is involved in regulation of metabolism and behavior through affecting the functioning of the hippocampus and amygdala^91^. The involvement of the brain hippocampus, as one part of the circuitry^25^, in regulating normoglycemia has been well established^26,92,93^. The hippocampus has connections to the hypothalamus, that is considered a part of the brain-centered glucoregulatory system^26,94,95^, but it is also a part of stress-related regulatory systems, such as HPA-axis^96^. Both of these systems affect blood glucose and insulin levels, but possibly through different circuitries. Thus, it is possible that the hippocampus is a mediator of these functionally related, but anatomically separate circuitries^97^. To conclude, it is possible that the developmental deficits in the hippocampus may be related to its ability to regulate glucose metabolism, and based on our results, the impairment of this regulation manifests near the onset of first psychosis.

### Hippocampus subfield morphology associates with insulin resistance in early psychosis

We found that higher insulin and insulin resistance associated statistically significantly with smaller hippocampal tail volume in FEP at one-year follow-up. Hereby, our result might indicate the bidirectional mechanism; the developmental and progressive deficits in the hippocampus may be related to its ability to regulate glucose metabolism, but also, that the dysfunctional hippocampus might further be more sensitive to the effects of glucose as the psychotic illness progress. This may lead to circular effect of progressive dysregulation of glucose metabolism, and increased volume loss in hippocampus in the progression of psychosis.

There is evidence that the anterior hippocampus is more strongly linked to the temporal pole and lateral temporal cortex, with only a small number of nerve fibres from these cortical areas projecting to the hippocampal tail. However, hippocampal tail has multiple connections to the primary visual cortex and the medial parietal cortex. These connections gradually decrease towards the head of the hippocampus^98^. Increased parietal and occipital lobe gyrification have been recently suggested to predict conversion to psychosis in clinical high-risk patients^99^. Gyrification index is considered a proxy marker for early cortical neurodevelopmental abnormalities^100,101^. These connections between hippocampal tail, parietal and occipital cortex, with possible different temporal manifestations in psychotic disorders, need to be studied further.

Widespread reductions in subfields, including hippocampal tail have been previously reported in type 2 diabetes, but not in prediabetes^34^. In our sample, fasting glucose values ranged from normal to prediabetic levels. Thus, it is possible that the mechanisms related to the tail volume defects associate with the worsening of glucose metabolism and are more related to the pathophysiology of psychotic illness itself, rather than to co-morbid diabetes. Also, these associations seem to emerge only after the onset of first psychosis. This association might also indirectly reflect other phenomena related to psychosis such as disruption of the blood brain barrier^102^. There is evidence of progressive volumetric changes of the hippocampal tail in chronic schizophrenia^23^, but also of a potential shared role of inflammation in insulin resistance and schizophrenia^103^. In our study, there was no difference in the associations between insulin and insulin resistance and tail volume while comparing first episode NAP and AP groups. Although the tail volume was smaller only in NAP compared to CTR, there was no significant changes in the tail volumes in FEPs in our longitudinal analyses. It is possible that progressive decline in tail volume associating with dysfunctional glucose metabolism may start after the first psychotic episode, but this might be related to a more chronic non-affective psychosis, or more specifically to chronic schizophrenia.

### Clinical outcome trajectories, hippocampus morphometry and glucose metabolism

We found associations between increased insulin or insulin resistance and impaired measures of clinical outcome, such as nonremission, poor daily functioning and transition to psychosis, in CHR. Hypothetically, this could be due to individual health behaviors, such as exercise and diet, which are subject to be influenced by symptomatology. However, a recent longitudinal study found worsening of fasting glucose levels in recent-onset psychosis, despite of improving lifestyle habits, clinical improvement and thus decreasing antipsychotic medication^44^. This might mean that deteriorating glucose metabolism is related rather to psychotic illness progression than poor health behaviors. Also, since we observed a worsening of insulin resistance near the onset of psychosis, it is possible, that the association between the deteriorating glucose metabolism and measures of outcome indicates that the metabolic processes seen in psychotic disorders progress during the prodromal period. This would support the current view of psychotic disorder as a systemic disease.

Notably, the FEPs with good level of functioning at follow-up had an increase in presubiculum volume. This might be evidence that the presubiculum is subject to state rather than trait influences, or it could alternatively be a result of therapeutic effects^104^, exposure to antipsychotic medication, or other confounding factors^105,106^. It is possible that the volume increases of the presubiculum are related to specific types of antipsychotic or antidepressant medication^107^. However, antipsychotic exposure or the use of antidepressants was not significantly associated with presubiculum morphology. Although our results of an association between presubiculum volumes and better functional outcome should be ultimately regarded as preliminary, it should be noted that they are in line with earlier reports of better GAF associating with bilateral increases^108^ or unchanged volumes^109^ of the hippocampus in FEP. In CHR, the results from previous studies of the associations between outcome and hippocampus volumes have been inconsistent^110^.

## Strengths and limitations

This study included characterizations of two clinical samples at baseline and follow-up, as well as across a one-year follow-up period. We used state-of-the-art methodology (FreeSurfer 7.1.1) for segmentation of hippocampus subfield volumes. We were also able to control for the confounding effects of medication by determining lifetime cumulative antipsychotic exposure and daily use of antidepressants. The clinical groups were compared to a random population sample control group. This study has two main limitations. Firstly, we were able to include only modest sample sizes. This is particularly true for the analyses including smaller subgroups of CHR-C, CHR-NC, NAP and AP. The follow-up period for determining the division into CHR-C and CHR-NC groups was adequate, but not optimal; the CHR-C group could possibly have been increased by extending the follow-up time. Secondly, the position of the internal boundaries between the hippocampal substructures segmented using scans with 1mm isometric voxel size relies heavily on prior knowledge from ex vivo training data. For this reason, the volumes of the internal subfields (dentate gyrus, molecular layer and CA4) must be interpreted with caution.

## Conclusions

Our longitudinal study suggests that higher fasting plasma insulin and insulin resistance associates with smaller hippocampal tail volumes in non-diabetic first-episode psychosis patients. Also, we found that insulin and insulin resistance worsened in CHR during the follow-up period. This effect was driven by high-risk patients converting to psychosis. In CHR, the glucometabolic deterioration was related to clinical outcome trajectories, such as transition to psychosis, non-remission, and poor level of functioning.

Our findings support the idea that psychotic disorders have heterogenous temporospatial subfield defects, but also common systemic manifestations near the onset of first psychosis, which could be relevant for understanding the etiology and progression of these disorders. Also, clinical attention should increasingly be diverted to the somatic health and metabolic state in the context of high clinical risk for psychosis and first episode of psychosis, as glucose metabolism appears to be related to both clinical outcome trajectories as well as hippocampal morphology in early psychosis.

## Supplementary information

Supplementary material

## Data Availability

The data supporting the findings of this study are available from the corresponding author upon reasonable request.

## Code availability

R code available upon request. All analyses were carried out with R version 4.2.1 (2022-06-23) “Funny-Looking Kid”^111^
