# Publisher Correction: Longitudinal study on hippocampal subfields and glucose metabolism in early psychosis

**DOI:** 10.1038/s41537-024-00495-9

**Published:** 2024-09-02

**Authors:** Reetta-Liina Armio, Heikki Laurikainen, Tuula Ilonen, Maija Walta, Elina Sormunen, Arvi Tolvanen, Raimo K. R. Salokangas, Nikolaos Koutsouleris, Lauri Tuominen, Jarmo Hietala

**Affiliations:** 1 PET Centre, Turku University Hospital, 20520 Turku, Finland; 2 Department of Psychiatry, University of Turku, 20700 Turku, Finland; 3 Department of Psychiatry, Turku University Hospital, 20520 Turku, Finland; 4 Department of Psychiatry and Psychotherapy, Ludwig-Maximilian University, D-80336 Munich, Germany; 5 The Royal’s Institute of Mental Health Research, University of Ottawa, Ottawa, ON Canada; 6 Department of Psychiatry, Faculty of Medicine, University of Ottawa, Ottawa, ON Canada

**Keywords:** Psychosis, Neuroscience, Psychosis

Correction to: *Schizophrenia* 10.1038/s41537-024-00475-z, published online 31 July 2024

In this article there was an error in Table 3(a), the value under the column “Estimated difference (mm^3^)” should be 87 instead of 870. The errors have been corrected in the HTML and PDF versions of the article.

**Corrected Table 3**

Table 3. a) Whole hippocampus volume pairwise between-group baseline comparison analysis of FEP, CHR and CTR, including age, sex, total intracranial volume and body mass index as covariates. b) Subregional post-hoc pairwise between-group baseline comparison analysis of FEP, CHR and CTR, including age, sex, total intracranial volume and BMI as covariates.

**Table:** 

a) Volume	Contrast baseline	Estimated difference (mm^3^)	95% Confidence Interval	DF	t-ratio	*p*	FDR corrected p	p with exposure*	FDR corrected p with exposure*
Whole hippocampus	CTR - CHR	87	−15.09 –189	202	1.680	0.0944	0.0944	0.1306	0.1306
Whole hippocampus	CTR - FEP	192	103.50–281	202	4.274	<0.0001	<0.0001	0.0012	0.0035
Whole hippocampus	CHR - FEP	105	3.37–207	202	2.037	0.0429	0.0644	0.1137	0.1306

b) Volume	Contrast baseline	Estimated difference (mm^3^)	95% Confidence Interval	DF	t-ratio	*p*	FDR corrected p	p with exposure*	FDR corrected p with exposure*
Tail	CTR - CHR	27.28	10.40–44.2	202	3.187	0.0017	0.0100	0.0022	0.0130
Tail	CTR - FEP	34.44	19.78–49.1	202	4.632	<0.0001	0.0002	0.0001	0.0017
Tail	CHR - FEP	7.16	−9.77–24.1	202	0.834	0.4053	0.4229	0.5466	0.5704
Presubiculum	CTR - CHR	10.58	−6.29–27.5	202	1.236	0.2177	0.3486	0.2562	0.4645
Presubiculum	CTR - FEP	21.56	6.90–36.2	202	2.900	0.0041	0.0199	0.0188	0.0904
Presubiculum	CHR - FEP	10.98	−5.96–27.9	202	1.278	0.2026	0.3486	0.3142	0.4645
Subiculum	CTR - CHR	8.90	−7.97–25.8	202	1.040	0.2995	0.3626	0.3604	0.4645
Subiculum	CTR - FEP	18.88	4.22–33.5	202	2.539	0.0119	0.0444	0.0442	0.1404
Subiculum	CHR - FEP	9.97	−6.96–26.9	202	1.161	0.2469	0.3486	0.3593	0.4645
CA1	CTR - CHR	10.20	−6.68–27.1	202	1.192	0.2347	0.3486	0.3215	0.4645
CA1	CTR - FEP	31.44	16.78–46.1	202	4.229	<0.0001	0.0004	0.0005	0.0062
CA1	CHR - FEP	21.24	4.31–38.2	202	2.473	0.0142	0.0444	0.0296	0.1184
Molecular Layer	CTR - CHR	10.72	−6.16–27.6	202	1.252	0.2119	0.3486	0.2573	0.4645
Molecular Layer	CTR - FEP	29.98	15.32–44.6	202	4.032	0.0001	0.0006	0.0008	0.0062
Molecular Layer	CHR - FEP	19.26	2.33–36.2	202	2.243	0.0260	0.0624	0.0527	0.1404
GCMLDG	CTR - CHR	7.91	−8.96–24.8	202	0.925	0.3563	0.3887	0.3871	0.4645
GCMLDG	CTR - FEP	18.28	3.62–32.9	202	2.458	0.0148	0.0444	0.0523	0.1404
GCMLDG	CHR - FEP	10.36	−6.57–27.3	202	1.207	0.2290	0.2486	0.3678	0.4645
CA2/3	CTR - CHR	4.26	−12.62–21.1	202	0.498	0.6194	0.6194	0.6887	0.6887
CA2/3	CTR - FEP	13.64	−1.02–28.3	202	1.835	0.0680	0.1484	0.1812	0.3955
CA2/3	CHR - FEP	9.38	−7.55–26.3	202	1.092	0.2759	0.3626	0.4186	0.4784
CA4	CTR - CHR	8.32	−8.56–25.2	202	0.972	0.3321	0.3796	0.3664	0.4645
CA4	CTR - FEP	17.2	2.54–31.9	202	2.314	0.0217	0.0578	0.0707	0.1695
CA4	CHR - FEP	8.88	−8.05–25.8	202	1.034	0.3022	0.3626	0.4580	0.4997

*CA* Cornu ammonis, *Molecular Layer* Molecular Layer of the CA fields and subiculum, *GCMLDG* The Granule Cell and Molecular Layer of the Dentate Gyrus, *FDR* False discovery rate.

*Total lifetime antipsychotic exposure (until baseline) included in the model.

*FEP* First-episode psychosis patient.

*CHR* Clinical high-risk patient.

*CTR* Population control.

*Total lifetime antipsychotic exposure included in the model.

