# Supplementary material for: Longitudinal study on hippocampal subfields and glucose metabolism in early psychosis
Source: Schizophrenia (Heidelb). 2024 Jul 31;10(1):66. doi: 10.1038/s41537-024-00475-z (PMC11291638; doi:10.1038/s41537-024-00475-z)
Supplement: Supplementary file 1 — Supplementary material [file 41537_2024_475_MOESM1_ESM.pdf]

ASSOCIATIONS BETWEEN ADJUSTED TOTAL HIPPOCAMPUS VOLUMES AND INSULIN RESISTANCE AT BASELINE (A) AND FOLLOW-UP (B,C)

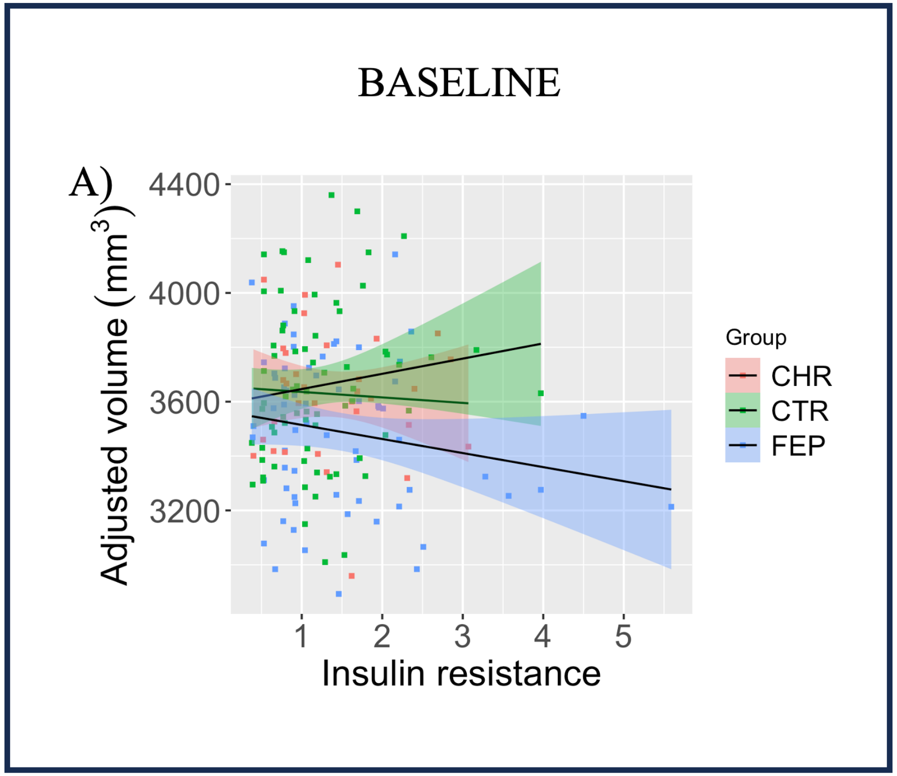

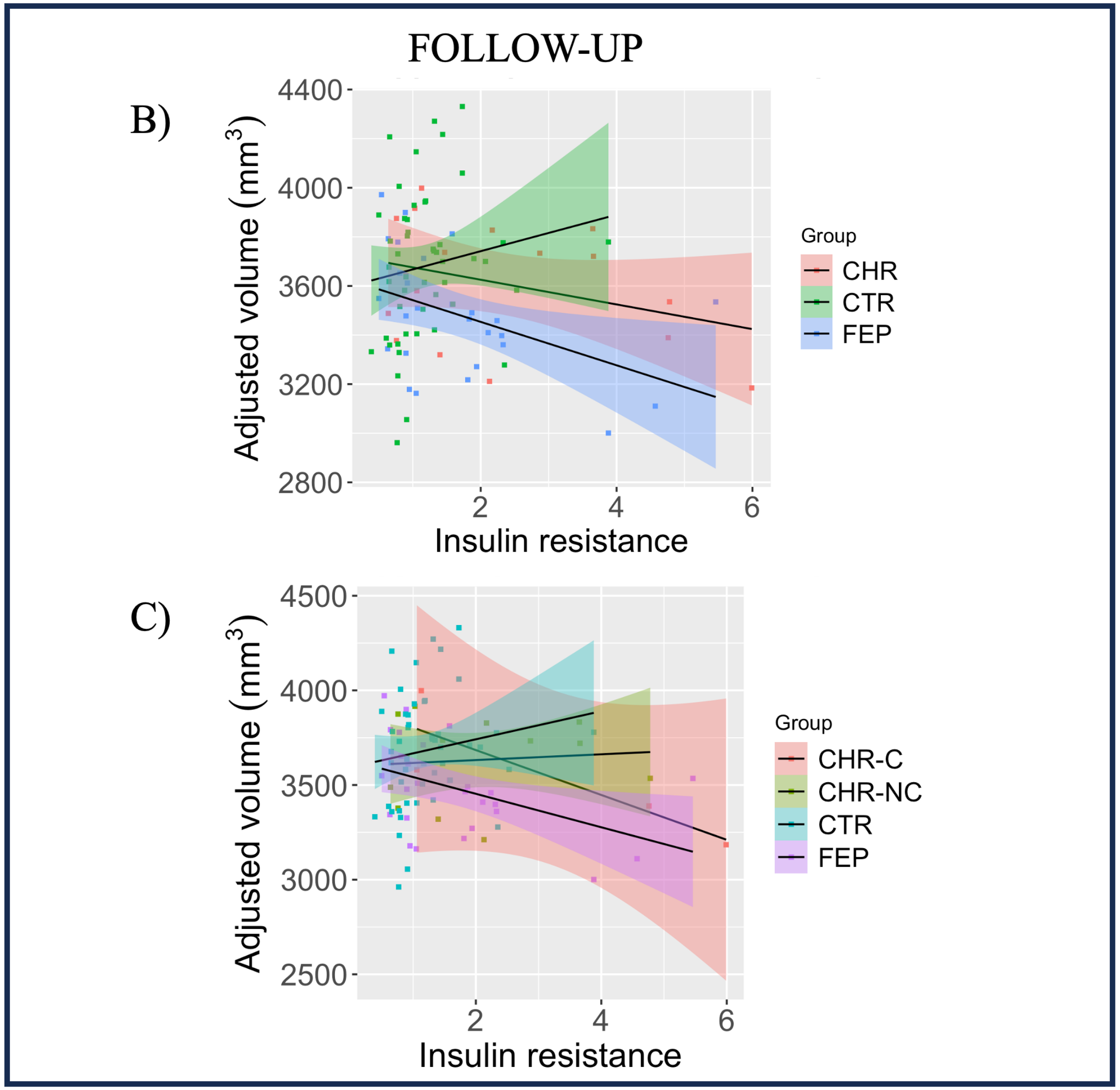

Supplementary figure 1. Associations between total hippocampus volumes and insulin resistance at baseline and follow-up
We observed a significant inverse association between insulin or insulin resistance and total hippocampal volumes in FEPs at the follow-up time point. Adjusting for lifetime exposure to antipsychotic medication or the use of antidepressants did not change these results. However, these results of total hippocampal volume did not survive the FDR correction.

FEP = First-episode psychosis patient, CHR-C = Clinical high-risk patients converting to psychosis,
CHR-NC = clinical high-risk patients not converting to psychosis during follow-up, CTR = population control group
The volumes are adjusted for age, sex, total intracranial volume and body mass index.
The line represents the association between the two variables, and the colored areas are confidence bands in each group.

Supplementary table 1. Psychotic and non-psychotic diagnoses of the participants.

**Diagnoses of non-affective psychoses defined according to DSM-IV:**	**Number**
Schizophrenia	8
Schizophreniform disorder	18
Schizoaffective disorder	4
Brief psychotic disorder	5
Delusional disorder	4
Psychosis not otherwise specified	17
**Diagnoses of affective psychoses defined according to DSM-IV:**	
Bipolar disorder with psychotic features	13
Major depressive disorder with psychotic features	9

**Subject**	**Non-psychotic diagnoses of general population controls defined according to DSM-IV:**
1	Panic disorder (lifetime, not past month)
2	Major depressive disorder (lifetime, not past month)
3	Social fobia (lifetime, past month)
4	Social fobia (lifetime, past month)
5	Specific fobia (lifetime, past month)
6	Depressive disorder not otherwise specified (lifetime)
7	Previous drug abuse (lifetime, not past month),<br>Panic disorder (lifetime, not past month)
8	Depressive disorder not otherwise specified (lifetime)

Supplementary table 2. Radiological exclusions of the study.

Subject	Radiological findings leading to exclusion:
1	Cortical dysplasia
2	Largely undeveloped areas of anterior and lateral parts of temporal lobe
3	Demyelinisation close to the insular cortex and lateral ventricles
4	Demyelinisation in the corpus callosum and insular cortex
5	Infarction or enlargement of perivascular space next to the lateral ventricles
6	Numerous subcortical white matter lesions
7	Focal lesion near hypohysis and infundibulum
8	Developmental enlargement of cerebrospinal fluid space

Supplementary table 3. Exploratory analyses

Exploratory analyses of associations between lifetime antipsychotic exposure or usage of antidepressant medication and hippocampal volumes were conducted. There was no significant association between lifetime antipsychotic exposure or the usage of antidepressant medication and hippocampal total or subfield volumetry in either FEP or CHR at the baseline or at the follow-up.

Further, there were no significant associations between antipsychotic exposure or the use of antidepressants and baseline hippocampal total or subfield volumes in CHR-C, CHR-NC or poor GAF, good GAF status, remission or non-remission status in FEPs or CHRs.

The information of the usage of antidepressive and mood stabilizing medication were collected:

Supplementary table 3.

ANTIDEPRESSANTS

	sertraline	venlafaxine	citalopram	escitalopram	paroxetine	bupropion	vortioxetine	mirtazapine
FEP baseline	1	4	6	11	0	1	0	2
CHR baseline	1	6	1	7	1	1	0	2
CTR baseline	0	0	0	0	0	0	0	0
FEP follow-up	0	0	2	3	0	1	1	2
CHR follow-up	0	6	0	4	1	0	0	4
CTR follow-up	0	0	0	0	0	0	0	0

MOOD STABILIZERS

	lithium	valproate	lamotrigine	gabapentin
FEP baseline	5	2	1	0
CHR baseline	0	0	2	0
CTR baseline	0	0	0	0
FEP follow-up	3	0	0	0
CHR follow-up	0	0	2	1
CTR follow-up	0	0	0	0

Supplementary table 4a Whole hippocampus volume pairwise between-group baseline comparison analysis of NAP, AP, CHR and CTR, including age, sex, total intracranial volume and body mass index as covar

Volume	Contrast baseline	Estimated difference (mm^3)	95 % Confidence Interval	DF	t-ratio	p	FDR corrected p	p with exposure*	FDR corrected p with exposure*
Whole hippocampus	CTR – CHR	88	-13.78–190	201	1.706	0.0896	0.1344	0.1255	0.2007
Whole hippocampus	CTR – AP	137	4.39–270	201	2.037	0.0430	0.0860	0.1338	0.2007
Whole hippocampus	CTR – NAP	215	117.30–313	201	4.336	<0.0001	0.0001	0.0006	0.0037
Whole hippocampus	CHR – AP	49	-94.64–192	201	0.672	0.5024	0.5024	0.7186	0.7186
Whole hippocampus	CHR – NAP	127	17.84–236	201	2.295	0.0228	0.0684	0.0617	0.1852
Whole hippocampus	AP – NAP	78	-62.66–218	201	1.093	0.2759	0.3310	0.2561	0.3073

**Total lifetime antipsychotic exposure included in the model*

Supplementary table 4b Post-hoc pairwise between-group comparison analysis of NAP, AP, CHR and CTR at baseline, including age, sex, total intracranial volume and body mass index as covariates.

Volume	Contrast baseline	Estimated difference (mm^3)	95 % Confidence Interval	DF	t-ratio	p	FDR corrected p	p with exposure*	FDR corrected p with exposure*
Tail	CTR – CHR	27.44	10.56–44.3	201	3.206	0.0016	0.0188	0.0021	0.02471
Tail	CTR – AP	15.53	-6.60–37.7	201	1.384	0.1679	0.4950	0.2621	0.54106
Tail	CTR – NAP	42.05	25.91–58.2	201	5.139	<0.0001	<0.0001	<.0001	0.00032
Tail	CHR – AP	-11.91	-35.74–11.9	201	-0.985	0.3259	0.4610	0.2567	0.54106
Tail	CHR – NAP	14.61	-3.54–32.8	201	1.587	0.1141	0.3650	0.1770	0.54106
Tail	AP – NAP	26.51	3.20–49.8	201	2.242	0.0260	0.1040	0.0245	0.15664
Presubiculum	CTR – CHR	10.74	-6.13–27.6	201	1.255	0.2108	0.4050	0.2500	0.54106
Presubiculum	CTR – AP	14.20	-7.93–36.3	201	1.265	0.2073	0.4050	0.3417	0.54106
Presubiculum	CTR – NAP	24.63	8.50–40.8	201	3.011	0.0029	0.0282	0.0119	0.11453
Presubiculum	CHR – AP	3.45	-20.38–27.3	201	0.286	0.7754	0.8090	0.9308	0.97123
Presubiculum	CHR – NAP	13.89	-4.26–32.0	201	1.509	0.1329	0.3990	0.2104	0.54106
Presubiculum	AP – NAP	10.44	-12.88–33.8	201	0.882	0.3786	0.4910	0.3628	0.54106
Subiculum	CTR – CHR	9.06	-7.81–25.9	201	1.059	0.2909	0.4610	0.3526	0.54106
Subiculum	CTR – AP	11.46	-10.68–33.6	201	1.021	0.3086	0.4610	0.4762	0.61782
Subiculum	CTR – NAP	21.97	5.84–38.1	201	2.685	0.0078	0.0628	0.0273	0.15664
Subiculum	CHR – AP	2.39	-21.45–26.2	201	0.198	0.8434	0.8610	0.9861	0.98611
Subiculum	CHR – NAP	12.91	-5.24–31.1	201	1.402	0.1624	0.4050	0.2417	0.54106
Subiculum	AP – NAP	10.52	-12.80–33.8	201	0.889	0.3749	0.4910	0.3592	0.54106
CA1	CTR – CHR	10.36	-6.51–27.2	201	1.211	0.2275	0.4020	0.3143	0.54106
CA1	CTR – AP	26.18	4.05–48.3	201	2.332	0.0207	0.0924	0.0555	0.24240
CA1	CTR – NAP	33.69	17.56–49.8	201	4.118	0.0001	0.0009	0.0006	0.00881
CA1	CHR – AP	15.82	-8.02–39.7	201	1.308	0.1923	0.4050	0.2697	0.54106
CA1	CHR – NAP	23.33	5.18–41.5	201	2.534	0.0120	0.0824	0.0237	0.15664
CA1	AP – NAP	7.51	-15.80–30.8	201	0.635	0.5259	0.6310	0.5061	0.63104
Molecular Layer	CTR – CHR	10.88	-6.00–27.8	201	1.271	0.2052	0.4050	0.2511	0.54106
Molecular Layer	CTR – AP	21.29	-0.84–43.4	201	1.897	0.0593	0.2190	0.1218	0.44957
Molecular Layer	CTR – NAP	33.58	17.44–49.7	201	4.104	0.0001	0.0009	0.0005	0.00881
Molecular Layer	CHR – AP	10.41	-13.43–34.3	201	0.861	0.3902	0.4930	0.5127	0.63104
Molecular Layer	CHR – NAP	22.70	4.55–40.8	201	2.466	0.0145	0.0833	0.0294	0.15664

Molecular Layer	AP – NAP	12.29	-11.03–35.6	201	1.039	0.3000	0.4610	0.2867	0.54106
GCMLDG	CTR – CHR	8.07	-8.80–25.0	201	0.943	0.3466	0.4750	0.3790	0.54106
GCMLDG	CTR – AP	14.47	-7.66–36.6	201	1.289	0.1987	0.4050	0.3312	0.54106
GCMLDG	CTR – NAP	19.95	3.82–36.1	201	2.439	0.0156	0.0833	0.0476	0.22864
GCMLDG	CHR – AP	6.40	-17.44–30.2	201	0.529	0.5973	0.6880	0.7659	0.83548
GCMLDG	CHR – NAP	11.88	-6.27–30.0	201	1.29	0.1984	0.4050	0.3141	0.54106
GCMLDG	AP – NAP	5.48	-17.84–28.8	201	0.463	0.6435	0.7020	0.6213	0.71003
CA2/3	CTR – CHR	4.42	-12.46–21.3	201	0.516	0.6062	0.6880	0.6779	0.75668
CA2/3	CTR – AP	14.52	-7.61–36.6	201	1.294	0.1973	0.4050	0.3395	0.54106
CA2/3	CTR – NAP	13.48	-2.66–29.6	201	1.647	0.1011	0.3470	0.2251	0.54106
CA2/3	CHR – AP	10.10	-13.74–33.9	201	0.835	0.4045	0.4980	0.5422	0.65064
CA2/3	CHR – NAP	9.06	-9.09–27.2	201	0.984	0.3263	0.4610	0.4671	0.61782
CA2/3	AP – NAP	-1.04	-24.36–22.3	201	-0.088	0.9299	0.9300	0.9565	0.97686
CA4	CTR – CHR	8.48	-8.39–25.4	201	0.991	0.3229	0.4610	0.3585	0.54106
CA4	CTR – AP	13.07	-9.06–35.2	201	1.164	0.2456	0.4360	0.3945	0.54106
CA4	CTR – NAP	19.01	2.88–35.1	201	2.323	0.0212	0.0924	0.0611	0.24456
CA4	CHR – AP	4.59	-19.25–28.4	201	0.379	0.7048	0.7520	0.8753	0.93366
CA4	CHR – NAP	10.53	-7.62–28.7	201	1.144	0.2541	0.4360	0.3834	0.54106
CA4	AP – NAP	5.94	-17.38–29.3	201	0.502	0.6160	0.6880	0.5943	0.69574

CA = cornu ammonis, Molecular Layer = Molecular Layer of the CA fields and subiculum, GCMLDG =The Granule Cell and Molecular Layer of the Dentate Gyrus, FDR = false discove
NAP = non-affective psychosis, AP = affective psychosis, CHR = clinical high risk for psychosis, CTR = population controls
